# Function of Hemoglobin-Based Oxygen Carriers: Determination of Methemoglobin Content by Spectral Extinction Measurements

**DOI:** 10.3390/ijms22041753

**Published:** 2021-02-10

**Authors:** Kathrin Smuda, Jonas Gienger, Philipp Hönicke, Jörg Neukammer

**Affiliations:** 1 Institute of Transfusion Medicine, Charité-Universitätsmedizin Berlin, 10117 Berlin, Germany; 2 Physikalisch-Technische Bundesanstalt, 10587 Berlin, Germany; jonas.gienger@ptb.de (J.G.); philipp.hoenicke@ptb.de (P.H.)

**Keywords:** hemoglobin-based oxygen carrier, artificial blood substitute, HbMP, methemoglobin determination, spectral extinction, spectral refractive index, sub-micrometer particle characterization, light scattering

## Abstract

Suspensions of hemoglobin microparticles (HbMPs) are promising tools as oxygen therapeutics. For the approval of clinical studies extensive characterization of these HbMPs with a size of about 750 nm is required regarding physical properties, function, pharmaco-kinetics and toxicology. The standard absorbance measurements in blood gas analyzers require dissolution of red blood cells which does not work for HbMP. Therefore, we have developed a robust and rapid optical method for the quality and functionality control of HbMPs. It allows simultaneous determination of the portion of the two states of hemoglobin oxygenated hemoglobin (oxyHb) and deoxygenated hemoglobin (deoxyHb) as well as the content of methemoglobin (metHb). Based on the measurement of collimated transmission spectra between 300 nm and 800 nm, the average extinction cross section of HbMPs is derived. A numerical method is applied to determine the composition of the HbMPs based on their wavelength-dependent refractive index (RI), which is a superposition of the three different states of Hb. Thus, light-scattering properties, including extinction cross sections can be simulated for different compositions and sizes. By comparison to measured spectra, the relative concentrations of oxyHb, deoxyHb, metHb are accessible. For validation of the optically determined composition of the HbMPs, we used X-ray fluorescence spectrometry for the ratio of Fe(II) (oxyHb/deoxyHb) and Fe(III) (metHb). High accuracy density measurements served to access heme-free proteins, size was determined by dynamic light scattering and analytical centrifugation and the shape of the HbMPs was visualized by electron and atomic force microscopy.

## 1. Introduction

Worldwide, about 85 million units of red blood cells are transfused annually [1] and an increasing need for blood is observed. However, the transfusion of red blood cells holds risks and limitations. Mismatched transfusions as well as transmissible infections present a major health hazard [2]. Additionally, the storage time of red blood cells is limited, cold chain principles must be respected and the logistics are highly demanding. Clearly, there is a highly and fast growing demand for an artificial oxygen carrier which is able to carry and deliver oxygen at the sites of need.

The search for an artificial blood substitute based on oxygen carriers has presented a great challenge in transfusion medicine for more than three decades. In the focus of research are hemoglobin-based oxygen carriers (HBOCs) [3]. However, clinical applications of such agents in humans is not yet possible due to several issues which need to be solved to ensure the patients’ safety. As free hemoglobin (Hb) induces strong vasoconstriction and hypertension [4], modified hemoglobin has been intensely investigated. Several modifications were based on intra- or intermolecular cross-linking or encapsulation. Diaspirin cross-linked hemoglobin (DCHb) or polymerized bovine hemoglobin showed potential for surgical patients in clinical trials, but also limitations caused by severe side effects like pulmonary hypertension and cardiac depression [5]. Oxygen oversupply due to low oxygen binding affinity and scavenging of nitric oxide (NO), respectively, might be the reasons [6]. Stroma-free hemoglobin passes the endothelial gaps of the capillary walls leading to nitric oxide binding. The bioavailability of NO is crucial to maintain homeostatic vascular function. It enables direct and indirect vasodilation and can be responsible for anti-thrombotic, anti-inflammatory and anti-proliferative effects [7]. Scavenging of NO can lead to vasoconstriction, thrombosis, inflammation, vascular hypertrophy and stenosis. Therefore, hemoglobin-based oxygen carriers should not be able to penetrate the endothelial gaps requiring that all their dimensions should exceed 100 nm [8]. Furthermore, clearance by phagocytes must be avoided. Hence, the dimensions of particles in a blood substitute must be smaller than 1 µm. It follows that particles in the submicron size range are the most promising approach.

Besides encapsulated Hb, loaded into lipid microvesicles [3], a promising method for fabrication of such hemoglobin particles is the co-precipitation–cross-linking–dissolution technique [8,9,10]. This method allows the entrapment of hemoglobin by co-precipitation with inorganic salts like manganese chloride (MnCl_2_) and sodium carbonate (Na_2_CO_3_) forming the Hb-containing template followed by a coverage with human serum albumin (HSA). Subsequently, hemoglobin and albumin molecules are cross-linked with glutaraldehyde. The dissolution of the template with ethylenediaminetetraacetic acid (EDTA) results in hemoglobin microparticles (HbMPs) with an average diameter of 700 nm with a narrow size distribution and a nearly uniform peanut-shaped morphology. The particles are able to bind and release oxygen. They show a low immunogenicity and no vasoconstrictive effects on afferent arterioles of mouse kidney glomeruli [10].

The approval of HBOCs for animal experiments and (pre-) clinical studies requires, besides investigations of toxicology and biocompatibility, also the characterization of the physical and physicochemical properties of the respective material. Morphological features of the particles, i.e. size, shape, density as well as the methemoglobin (metHb), oxyhemoglobin (oxyHb), deoxyhemoglobin (deoxyHb) amounts and the oxygen binding capacity need to be determined. It is important that these quantities are accessible for quality control by a rapid and reliable method. This demand cannot be met by standard procedures applied in laboratory medicine. In particular, HbMPs cannot be lysed like erythrocytes, photometric measurements are disturbed by light scattering, oxygen release measurements are time consuming and the deoxyHb content cannot be determined at present. To address this gap and to establish a rapid and reliable characterization of suspensions of HbMPs, we apply spectral transmission measurement and data analysis and present results in this paper. Our method, designated as spectral extinction measurement in particle suspensions and analysis (SEMPA) was recently demonstrated to allow the determination of size and refractive index of sphered erythrocytes [11]. By selecting the appropriate model for light scattering, i.e. Lorenz-Mie theory [12] for spherical particles or the T-matrix [13] method for small non-spherical particles with high symmetries, the ensemble averaged spectral extinction cross section of the particles is calculated. This measurand, i.e. the quantity to be measured [14], sensitively depends on the complex refractive index (RI) [15] and hence on the composition of the particles investigated. In our work, we analyze spectral extinction cross sections to determine the content of metHb, oxyHb as well as heme-free globins (Gl)/human serum albumin (HSA) of a specific batch of HbMPs.

For the validation of our spectral extinction measurements, we applied near edge X-ray absorption fine structure (NEXAFS) fluorescence measurements, being sensitive to the ratio of Fe(II) and Fe(III) thus allowing the determination of the relative concentrations of functional sum of oxyHb and deoxyHb and metHb. Alternatively, as demonstrated recently, the metHb fraction of the total Hb content and the total Hb concentration are accessible by NMR relaxometry [16].

Our investigations were complemented to support the analyses of spectral extinction measurements and interpretation of results. For this purpose, we applied scanning electron microscopy (SEM), atomic force microscopy (AFM), dynamic light scattering (DLS), analytical centrifugation (AC), flow cytometric measurements (FCM), high accuracy density measurement and spectrophotometric determination of the hemoglobin concentration after previous enzymatic digestion. In addition, the packed particle volume (PPV) was determined accounting for the elastic deformability of the HbMP.

The fundamental advantage of our approach is that intact microparticles are analyzed in suspension by extinction measurements. The straightforward access to the essential features of the particle ensemble stands in contrast to presently applied indirect methods. In particular, the ratio of oxyHb/deoxyHb as well as the content of metHb are not accessible by such indirect measurements, but can now be determined by SEMPA, since oxygenation and deoxygenation can be reversibly carried out.

We expect that our method can be used for the quantification of the function of various particles based HBOCs and possibly contribute to the optimization of their fabrication thus facilitating the initiation of pre-clinical studies.

## 2. Results and Discussion

### 2.1. Fabrication of Hemoglobin Microparticles

**Figure 1:**
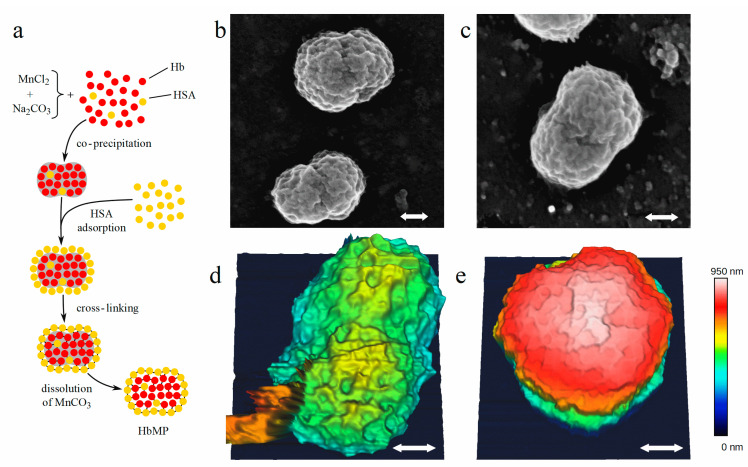
(**a**) Production scheme of HbMPs by co-precipitation and cross linking. (**b**,**c**) Images of HbMPs taken by scanning electron microscopy. (**d**,**e**) Atomic force microscopy of HbMPs attached with two different orientations to the surface. The white arrows correspond to a length of 200 nm in each image.

Hemoglobin microparticles particles (HbMPs) were fabricated as described previously [10], a schematic of the production process is illustrated in Figure 1a. Briefly, bovine hemoglobin (10 mg/mL) was entrapped by co-precipitation of 0.25 mol L^−1^ manganese chloride (MnCl_2_) and 0.25 mol L^−1^ sodium carbonate (Na_2_CO_3_) and the resulting particles are covered with human serum albumin (HSA). Hemoglobin and albumin molecules were cross-linked by glutaraldehyde with a final concentration of 0.04%. and the salt templates were dissolved by 0.18 mol L^−1^ EDTA resulting in the final HbMPs.

### 2.2. Morphology of Hemoglobin Microparticles

To obtain access to the shape of the particles and to estimate the size, we applied scanning electron microscopy (SEM) and atomic force microscopy (AFM). As is evident from Figure 1b–e the particles exhibit a non-spherical, peanut like shape with a ratio between the short and long axes of approximately 1:1.5. Inspection of SEM and AFM images yields a range between 600 nm and 1100 nm for the long axis of the particles. Our observations show that the size of the particles derived from SEM is smaller compared to AFM, which is explained by the different preparation procedures. For SEM, the samples must be dried and the particles are covered by a thin platinum layer. Hence, due to dehumidification shrinking of the HbMPs might be induced [17]. On the other hand, AFM images are taken in solution approximately matching the biological environment for the intended application. The particles in Figure 1d,e, imaged by AFM, were oriented differently, one with its long axis parallel (Figure 1d) and the other with the particle’s long axis upright (Figure 1e) with respect to the surface. Due to their different orientation, the maximum heights (encoded in false colors) of the particles relative to the surface, are approximately 600 nm and 900 nm, respectively. In SEM and AFM images, the waist of the particles is visible. Furthermore, both methods reveal the fine structure of the surface, which is associated with the MnCO_3_ scaffold used to incorporate the proteins and which is finally removed when producing the HbMPs.

### 2.3. Optical Determination of metHb in Hemoglobin Microparticles

To measure collimated transmittance spectra of diluted HbMP suspensions, we used a dedicated optical setup [11] (Figure S1), described in more detail in the Materials and Methods section. Regular spectrophotometers are intended for purely absorbing samples but unsuitable for the quantitative analysis of light-scattering samples. In contrast, our setup allows the quantitative determination of the spectral extinction cross section of the HbMPs in suspension, since unwanted contributions from light scattered in non-forward directions are negligible due to the small divergence of the incident light beam and a low angle of detection for the transmitted light.

We derived from the measured spectral transmittance T(λ) of the particle ensemble the average spectral extinction cross section ${\bar{C}}_{\mathrm{ext}}\left(\lambda \right)$ according to:

(1) $${\bar{C}}_{\mathrm{ext}}\left(\lambda \right)=\frac{-1}{\ell \;\;C_{0\;\;}\varphi }\;\ln\;T\left(\lambda \right)$$

where $C_{0}$ denotes the concentration of HbMPs in the stock suspension, φ the volume fraction of the stock suspension in the measurement suspension and ℓ the absorption length of the cuvette.

In order to avoid the influence of large uncertainties associated with the direct flow cytometric measurement of the concentration $C_{0}$ and the values derived from particle size and the packed particle volume (PPV) (see Section 2.5. Physical Properties of HbMPs), for quantitative comparison we consider the quantity:

(2) $$Z\left(\lambda \right)=\frac{{\bar{C}}_{\mathrm{ext}}\left(\lambda \right)}{\bar{V}}=\frac{-1}{\ell \;\;\mathrm{PPV}\;\;\varphi }\;\ln\;T\left(\lambda \right)$$

being the volume-specific extinction cross section (VSECS). The symbol $\bar{V}$ is the mean volume of the HbMPs and PPV, i.e. packed particle volume denotes the solid fraction of particles in the stock solution. The advantage of our approach is that the PPV is known to higher accuracy than the particle concentration in the stock suspension. Furthermore, compared to ${\bar{C}}_{\mathrm{ext}}\left(\lambda \right)$, Z(λ) changes only moderately with the mean particle size (Figures S2 and S3), while being equally sensitive to the relative Hb composition.

**Figure 2:**
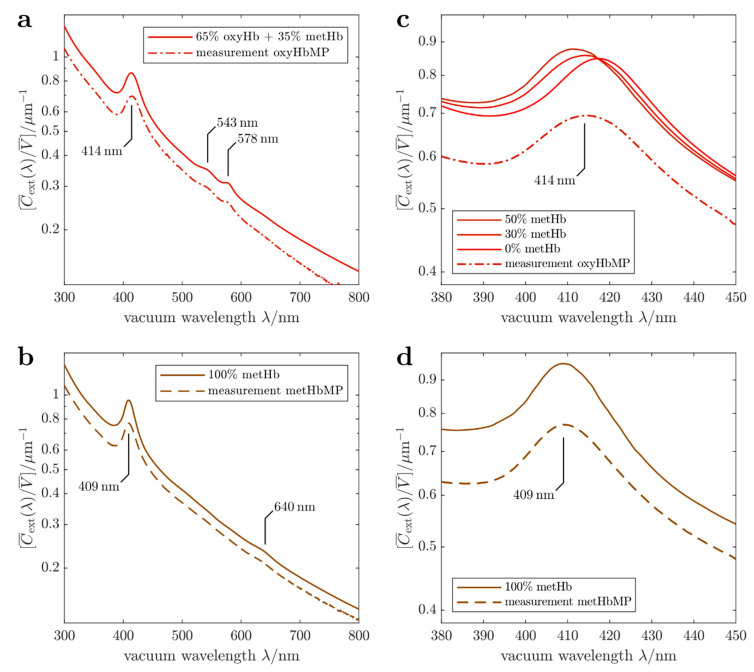
Results of spectral extinction measurements and simulations. (**a**) Volume-specific spectral extinction cross sections measured for oxyHbMP (dot-dashed red line) and calculated (red line) using 35% fraction of metHb and 65% oxyHb. (**b**) Volume-specific spectral extinction cross sections measured for metHbMP (dashed brown line) and calculated for 100% metHbMP (brown line). (**c**) Comparison of the measured spectral cross section in the Soret band and cross sections calculated for various metHb fractions. (**d**) Reduced wavelength range to show the Soret band of the measured and calculated extinction spectra.

**Figure 3:**
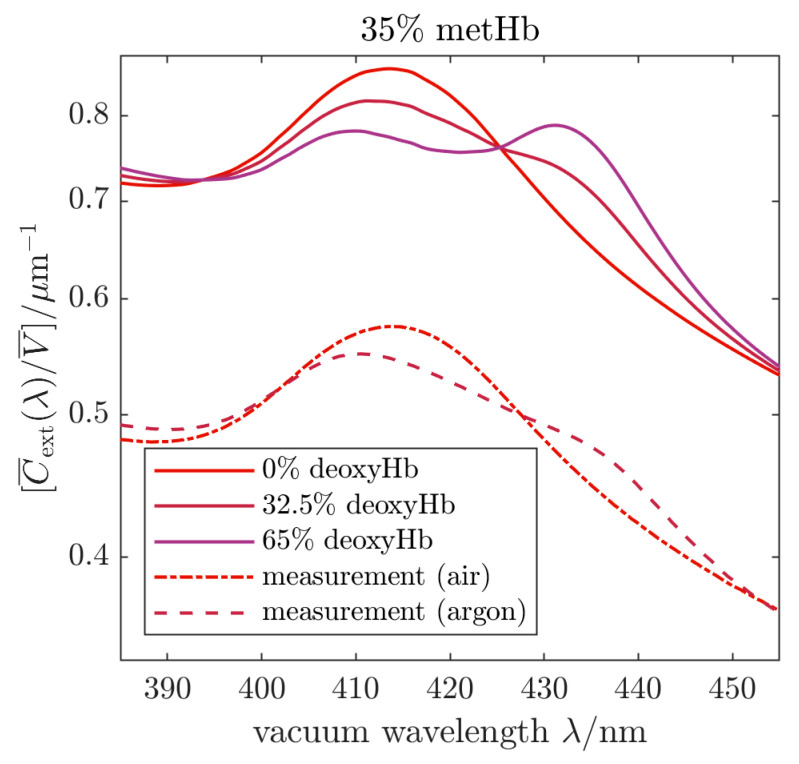
Volume-specific spectral extinction cross sections measured (dash-dotted and dashed lines) in air and using argon for deoxygenation. Calculated, volume-specific spectral extinction cross sections (solid lines) are included to illustrate the change of oxygenation of the HbMPs.

Experimental results and calculated extinction spectra are compared for three differently treated samples: (a) exposed to air and hence saturated with oxygen, (b) treated with sodium nitrite (NaNO_2_) to convert all Hb components to metHb and (c) purged with argon to obtain deoxygenated Hb. These preparations are referred to in Figure 2 and in the following as (a) oxyHbMP and (b) metHbMP. The case (c) is shown in Figure 3 as “measurement (argon)”. Calculations are based on the Lorenz-Mie theory [12,15] taking into account the known optical properties of the individual proteins in the HbMPs (see Section 3.6. Model for optical properties). Lorenz-Mie theory refers to the exact mathematical solution for the scattering and absorption of light by a homogeneous spherical particle. Its use is justified here because the influence of the HbMPs’ non-spherical shape on the extinction spectra was proven to be negligible [18].

In Figure 2a, the measured extinction cross section of oxyHbMP reveals the decrease of the cross section with increasing wavelength, caused by light scattering as well as the absorption bands of hemoglobin centered at about 413 nm (Soret band), 543 nm and 578 nm (Q-band). The corresponding calculated cross section was varied with respect to the ratio of oxyHb and metHb and compared to measured spectra. The resulting simulated curve in Figure 2a, obtained for 35% metHb abundance relative to the total Hb, does reflect the same behavior as the measured one, except the shift to higher values of the volume-specific cross section Z(λ). We attribute this deviation in the smaller measured cross sections, which were observed in all measurements, to systematic uncertainties caused: (i) by particle loss due to adhesion when pipetting, (ii) by agglomeration, (iii) or by trapped volume leading to a systematic increase in the PPV value. However, the observed spectral characteristic is well reproduced by our simulations. In addition, based on our simulations we estimated an accuracy for the determination of the metHb fraction to be 5 percentage points, i.e. the uncertainty is given by $u\left(\phi_{\mathrm{metHb}}\right)=0.05$, where $\phi_{\mathrm{metHb}}$ is the mass fraction of metHb relative to the total Hb. The spectrum measured for 100% metHb and the related calculated spectral cross section are shown in Figure 2b. For calibration purposes, the Hb in the HbMP suspension was completely converted to metHb by NaNO_2_. Compared to the oxygenated sample, the Soret band is slightly shifted from 413 nm to about 409 nm and its width is somewhat reduced. In addition, the Q bands characteristic for oxyHb are missing and a small metHb specific peak appears at approximately 640 nm. Again, the observed spectral features are reproduced by the cross section simulated for 100% metHb portion. In Figure 2c,d an enlarged view for the wavelength region of the Soret bands is shown. We include in Figure 2c results for calculated cross sections assuming different metHb fraction, i.e. 0%, 30% and 50%. The simulated curve for 100% metHb is shown in Figure 2d for comparison with the experiment. It is obvious from our simulations and measurements that the increase of the metHb portion results in a shift of the Soret band towards smaller wavelengths. The best agreement between measured oxygen saturated HbMP suspensions was obtained for a metHb fraction of 35% and 65% oxyHb.

Besides the samples of HbMPs saturated with oxygen and converted to metHbMP, we studied the transition between oxygenated HbMPs and partially deoxygenated HbMPs. Starting with the fully oxygenated sample as shown in Figure 2a,c, i.e. the fractions correspond to 65% oxyHb, 35% metHb and 0% deoxyHb, the cuvette was flushed with argon for about 60 min. As can be seen from Figure 3 the experimental cross section changes when the sample is exposed to argon. Besides the Soret band characteristic for oxyHb, we observe a superposition with the absorbance of deoxyHb, the maximum of which is located at about 430 nm. Deoxygenation using argon is difficult and complete release of oxygen requires an unrealistically long treatment of the sample. Hence, only partial deoxygenation was reached in our experiment and the agreement with the simulation result is best for 32.5% deoxyHb, 32.5% oxyHb and 35% metHb. To illustrate the change of the line shape, we include also calculations for 0% deoxyHb and 65% deoxyHb in Figure 2e. In particular, for 0% deoxyHb the combination of the metHb and oxyHb bands leads to a slight shift and a broadening of the measured absorption band. On the other hand, for 65% deoxyHb both Soret bands for the respective Hb variants, i.e. metHb and deoxyHb, are clearly discernable.

### 2.4. MetHb Detection by X-ray Fluorescence

Near edge X-ray absorption fine structure (NEXAFS) is sensitive for the chemical state of the targeted element. Hence, due to the different oxidation states of iron Fe(II) and Fe(III) in oxyHb/deoxyHb and metHb, both variants are distinguishable in the HbMP suspension by scanning the photon energy of the exciting radiation across the L_2_ and L_3_ absorption edges of iron. The scanning range between 698 to 736 eV corresponds to a range of vacuum wavelengths between 1.776 to 1.685 nm.

**Figure 4:**
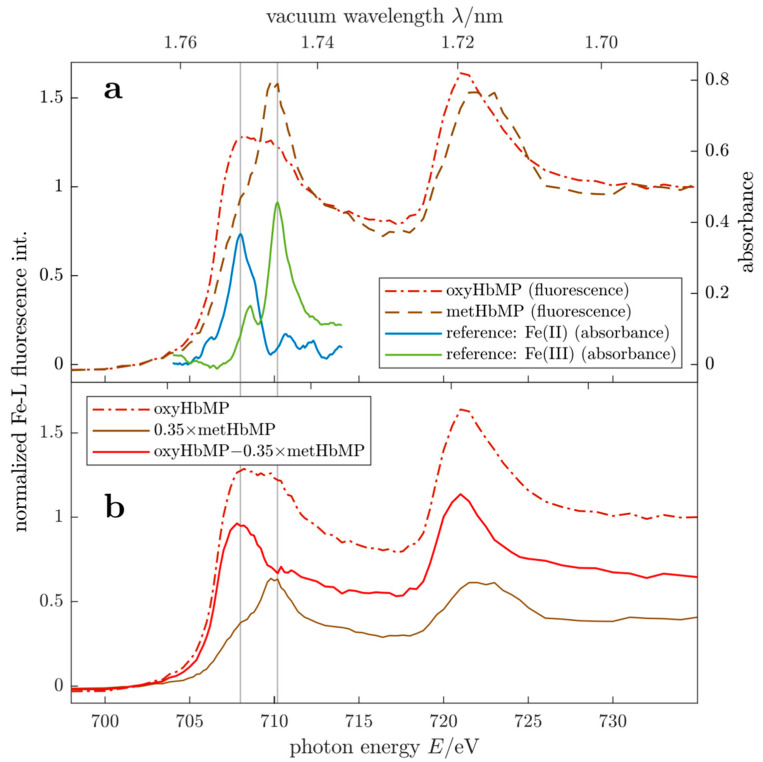
(**a**) NEXAFS fluorescence spectra of an oxygenated HbMP suspension (dot-dashed red trace) and metHbMP (dashed brown graph). For comparison, the absorbance of reference spectra for Fe(II) and Fe(III) [18] are included as blue and green lines, respectively. (**b**) Analysis of the oxygenated HbMPs (dot-dashed red curve) spectra as superposition of HbMPs containing 35% metHb and 65% oxyHb.

In Figure 4a NEXAFS fluorescence spectra of two HbMP suspensions are shown, exposed to air (dot-dashed red curve, oxyHbMP) or treated with sodium nitrite (NaNO_2_) to convert all Hb variants to metHb (dashed brown trace). For metHbMP, apart from a small shoulder at the low energy edge, the spectrum is dominated by the peak caused by the Fe(III) absorbance. Hence, approximately all Hb variants are converted to metHb. We used this measurement for normalization at 100% metHb content. The spectrum of the oxyHbMP shows a broad absorbance feature between 705 eV and 715 eV with the indication of two superposed maxima. It is evident that these maxima in the oxyHbMP spectrum coincide with the peaks of the Fe(II) and Fe(III) absorbance in the reference spectra [19]. To guide the eye, we include vertical lines at the corresponding energies. These two contributions to the measured oxyHbMP fluorescence spectrum are illustrated in Figure 4b. To prove the consistency with results of optical extinction measurements, the measured metHbMP spectrum was scaled according to the 35% metHb content in the oxyHbMP sample (the result is included as brown trace). The resulting difference spectrum (red line) between the measured oxyHbMP fluorescence and the scaled metHbMP exhibits a single Fe(II) peak only, thus being in agreement with the Fe(II) and confirming that the X-ray fluorescence spectra are in accordance with results derived by optical transmission measurements.

### 2.5. Physical Properties of Hemoglobin Microparticles

To set the size distribution for the analysis of the spectral extinction measurements, we characterized the HbMP sample with respect to the (sphere-equivalent) particle volume, the particle size distribution and the packed particle volume (PPV). In addition, the particle concentration in the stock suspension was determined directly by flow cytometry and derived from the measurements of the particle size and the PPV. Densities of the HbMP stock suspension and the supernatant were measured and used to calculate the density of the HbMP. Densities of reagents used for sample dilution and preparation, i.e. acetated Ringer’s solution and Pronase solution were determined. All values and estimated uncertainties are listed in Table 1 together with density increments of heme-free globins/HSA and bovine Hb, needed to calculate the density of the HbMP. Details of the different methods applied for the characterization of the HbMP are given in the corresponding paragraphs in Section Materials and Methods, i.e. dynamic light scattering (DLS), analytical centrifugation (AC), determination of the PPV, flow cytometry and density measurements.

The results for the median particle diameter and the relative distribution width differ for the DLS and AC methods. As previously discussed [20], the determination of particle size in the 10 to 1000 nm range is complex and the results strongly depend on the specific method used. In particular, polydisperse samples like the HbMPs are sensitive to such method specific differences. In addition, elastic properties, porosity and orientation of the HbMPs will influence the determination of size and size distribution. Taking into account that these effects are more pronounced in AC measurements due to the high centrifugation forces, for our analysis of spectral extinction measurements, we rely on the DLS results.

In order to calculate the concentration of the particles in the HbMP stock suspension, the total Hb concentration in the HbMPs and their density, we measured the packed particle volume. The details of the calculations and the PPV measurement are given in the section Materials and Methods. In principle, the PPV is determined similar to hematocrit measurements in blood samples. However, due to the smaller size and lower density of the HbMPs compared to erythrocytes [21], we increased the relative centrifugal force and the centrifugation time to assure complete sedimentation. To account for the elastic deformability of the HbMPs, the boundary between the solid fraction and the supernatant was read out several times to obtain the final value after about 100 h when relaxation of the particles was complete (Figure S4). We observed a change of the PPV value of 4%, the end point is given in Table 1. In our analyses, volume of fluid trapped between the solid phase particle is neglected, hence the value given corresponds to an upper limit.

The particle concentration is calculated using the PPV and the sphere-equivalent volumes determined by DLS and AC. Since the different–method specific–sizes result in correspondingly different concentrations, we used flow cytometry to directly measure the concentration of HbMPs in the stock suspension. However, flow cytometry yields the lowest concentration associated with particle loss due to adhesion on tube and container walls during preparation and measurement and agglomeration of the HbMPs. Taking into account the respective uncertainties, it follows from our observations that at present only a concentration range of 260 pL^−1^ $\leq C_{0}\leq$ 1125 pL^−1^ can be reliably given. As consequence, we introduce the volume-specific extinction cross section Z(λ), defined in Equation (2), which does not explicitly depend on the concentration and only slightly changes with particle size.

In our analysis of the optical extinction spectra, we noted that the experimental data are not well reproduced by the simulations, if the HbMPs are modeled using only the three Hb components for the complex RI. The simulated cross sections are too low under this assumption. Instead, a significant content of heme-free proteins had to be included in the model. Such proteins do not exhibit significant absorption features in the spectral range under consideration, but increase the real part of the RI and hence influence the light-scattering properties, including extinction cross sections. While a certain content of HSA in the HbMPs (≤10% of the total protein content) is expected from the production, the apparent concentration of heme-free proteins is much higher. We attribute this to Hb molecules that lose their heme group during production or storage of the particles, i.e., globin molecules. Throughout the text, the heme-free proteins in the HbMP are referred to as a globin/human serum albumin mixture (Gl/HSA). Their content was assessed by means of high accuracy density measurements.

The densities of the stock suspension of HbMPs and the supernatant were measured by means of an instrument based on a mechanical oscillator method [22]. In addition, the densities of acetated Ringer’s solution used for diluting the stock suspension for AC measurements and Pronase solution for the enzymatic digestions in context with the spectrophotometric total Hb determination are listed in Table 1. The value for ultrapure water that served for validation is also included. The uncertainties of typically 10^−4^ g mL^−1^ represent standard deviations for ≥10 repeat measurements. In analogy with the determination of the density of erythrocytes [20], we obtain the ensemble averaged density of the HbMPs from the measured densities of the HbMP stock suspension $\rho_{}^{sus}$ and the supernatant $\rho_{}^{sup}$ in combination with the PPV value (see Section 3.12. Density of HbMPs) according to:

(3) $$\rho_{}^{HbMP}=\frac{1}{\mathrm{PPV}}\;\left(\;\rho_{}^{sus}-\;\;\;\rho_{}^{sup}\;\right)+\rho_{}^{sup}$$

As described in the Hb Concentration in HbMPs subsection, the density difference $\rho_{}^{sus}-\rho_{}^{RAc}$ derived from these measurements is compared to calculated values based on the linear superposition of the mass concentrations $\beta_{\mathrm{Hb}}^{sus}$ of Hb and $\beta_{\mathrm{Hb}}^{sus}$ of heme-free globin/HSA (Table 2), weighted by the corresponding density increments DI (see Table 1):

(4) $$\rho_{}^{sus}-\rho_{}^{RAc}={\mathrm{DI}}_{\mathrm{Hb}}^{}\;\;\beta_{\mathrm{Hb}}^{sus}+{\mathrm{DI}}_{\mathrm{Gl}/\mathrm{HSA}}^{}\;\;\beta_{\mathrm{Gl}/\mathrm{HSA}}^{sus}$$

This comparison reveals that the density difference $\rho_{}^{sus}-\rho_{}^{RAc}$ cannot be explained by a particle consisting of predominantly hemoglobin and small HSA contributions. A significant mass of heme-free globin must be present. It follows from the absolute mass concentrations given in Table 2 that the suspension–and most likely also the HbMPs − contains 48% hemoglobin and 52% heme-free globin/HSA. These results derived from the high accuracy density measurement explain the low Hb concentrations in the particle suspension and in the HbMPs.

### 2.6. MetHb and Functional Hb in Hemoglobin Microparticles

Results for the metHb fraction in HbMPs obtained by spectral extinction measurements and NEXAFS are compared to measurements based on oxygen release [23,24,25], details of which are given in Materials and Methods section. The absolute mass concentrations for the functional Hb components determined by the oxygen release procedure are related to the total Hb concentrations of the stock suspension, listed in Table 2a. The total Hb concentration was obtained by spectrophotometric absorbance measurements. Briefly, HbMPs were enzymatically digested followed by the alkaline haematin and detergent (AHD) conversion procedure [26,27]. In addition, in Table 2a the values for the Hb mass concentrations in the supernatant of the HbMP stock solution and in the HbMP are given as well as the concentration of heme-free proteins and HSA. As described in Materials and Methods, these values are derived from the measured total Hb mass concentration, high accuracy density measurements and the packed particle volume (PPV), i.e. the solid fraction in the HbMP stock suspension (see Table 1). The total mass concentrations summarized in Table 1a elucidate that about 8% of the hemoglobin is not incorporated in the particles or is released during production or storage into the supernatant. The amount of heme-free globins/HSA slightly exceeds 50%.

The part of functional Hb is listed in Table 2b for the three different preparations of the sample referred to as oxyHbMP for the suspension exposed to air and thus saturated with oxygen, as metHbMP when treated with sodium nitrite (NaNO_2_) and deoxyHbMP when using sodium dithionite (Na_2_S_2_O_4_). The oxygen release method allows the determination of absolute values for the functional hemoglobin, for the air-equilibrated suspension the mass concentration is 11.8 g L^−1^. As can be seen from the first column in Table 2b, for the deoxygenated sample as well as for the metHbMP suspension, we still observe a significant oxygen release, indicating that the conversions were not complete. The absolute values were related to the total hemoglobin concentration in the sample (Table 2a) and we obtain the relative concentrations of non-functional hemoglobin and metHb. In this work, we focus on the comparison of the relative concentrations of the non-functional component for oxygenated samples (Table 2b, yellow row). The metHbMP suspensions were used for the validation of the spectral extinction and X-ray fluorescence methods, as indicated by the value set to 100%.

It is apparent that the concentration of metHb determined by the oxygen release procedure as difference between the total Hb concentration and the oxyHb concentration is significantly larger compared to the results obtained using spectral extinction and X-ray fluorescence measurements. This discrepancy is attributed to the hindered confirmation change of hemoglobin when embedded in the salt matrix and cross-linked, which results in increased time constants for oxygen intake and release and change in the reaction equilibrium. On the other hand, the optical method distinguishes metHb and oxyHb on the basis of different refractive indices and X-ray spectroscopy is sensitive against Fe(II) and Fe(III), independent on the localization of the two Hb components. The value of 35% determined for the relative metHb concentration corresponds to an absolute value of 0.65 x (120.1) g L^−1^ ≈ 78 g L^−1^ of functional hemoglobin in the particles. Taking into account the ratio of Hb and heme-free globin/HSA in the stock suspension of 1.086, we obtain a total protein concentration of (120.1 + 130.5) g L^−1^ for the particles, which results in a fraction of about 31% of functional Hb in the particles. This value obtained for the specific batch investigated indicates that it is absolutely necessary to control the production process in order to improve the oxygen transport capacity and to allow reproducible fabrication of the HbMPs.

## 3. Materials and Methods

### 3.1. Experimental Designs

The objective of this work was to develop a robust and reliable method with a low turnaround time to determine the biological function of HbMPs as promising candidate for artificial blood substitute. The function of HbMPs is defined by a sufficiently high hemoglobin component capable for transport of oxygen, while the methemoglobin component is required to be as low as possible. To quantify the abundance of oxyHb, deoxyHb and metHb in the HbMPs, we used a dedicated optical setup (Figure S1) [11] to measure collimated transmittance spectra of diluted HbMP suspensions. In contrast to a regular spectrophotometer, intended for purely absorbing samples but unsuitable for the quantitative analysis of light-scattering samples, our setup, described in Subsection Optical Setup, features a very low angle of detection for the transmitted light. This allows the quantitative determination of the spectral extinction of the HbMPs in suspension without unwanted contributions from light scattered in non-forward directions. The results of the optical extinction measurements were validated by comparison with other methods sensitive to the composition and function of the HbMPs, i.e. high accuracy density determination, NEXAFS fluorescence and oxygen release determination.

To validate our measurements and analyses, three different modifications of hemoglobin microparticles were prepared. Particle suspensions, exposed to filtrated air when preparing the suitable dilution for the respective measurement are saturated with oxygen and hence identified as oxyHbMP. Samples containing deoxygenated particles, marked as deoxyHbMP, were generated with a solution containing 2 mg/mL sodium dithionite (Na_2_S_2_O_4_). Extinction spectra (Figure 3) of deoxyHbMP were obtained by flushing the diluted measurement suspension with argon. Hemoglobin in the HbMPs was converted into metHb by sodium nitrite (NaNO_2_) with final concentrations of 10 mM NaNO_2_ and 10% HbMPs, the samples were labelled as metHbMP.

### 3.2. Materials

Bovine hemoglobin, derived from fresh whole blood (Biophyll GmbH, Dietersburg, Germany) by hypertonic hemolysis [28] was used for the production of the HbMPs. Glutaraldehyde, manganese chloride (MnCl_2_) tetrahydrate, sodium carbonate (Na_2_CO_3_), sodium nitrite (NaNO_2_), phosphate-buffered saline pH 7.4 were purchased from Sigma-Aldrich (Munich, Germany). EDTA and sodium dithionite (Na_2_S_2_O_4_) were provided by Fluka (Seelze, Germany) and sodium hydroxide (NaOH) by Carl Roth (Karlsruhe, Germany). Ampuwa and sterile 0.9% NaCl solution were purchased from Fresenius Kabi Deutschland GmbH (Bad Homburg, Germany). Human serum albumin solution 20% was obtained from Grifols Deutschland GmbH (Frankfurt am Main, Germany) and Pronase purchased from Sigma-Aldrich Chemie GmbH (Munich, Germany). The fabricated suspension of HbMPs was aliquoted in 15 mL vials under sterile conditions and stored at 4 °C. The volumes required for the various measurements were also taken under sterile conditions.

### 3.3. Morphology of Hemoglobin Microparticles

The shape of the particles was determined by scanning electron microscopy (SEM) and atomic force microscopy (AFM). Whereas for SEM extensive preparation is required and dry samples are examined, HbMPs in suspension are investigated by AFM.

For scanning electron microscopy, a Leo Supra 35 VP microscope (Zeiss, Oberkochen, Germany) was used. The suspension was pre-diluted to a PPV of 2% and mixed for about 5 min using a tube roller, thereafter treated in an ultrasonic bath at 35 kHz and 130 kHz for 15 min and 5 min, respectively. First investigations were based on positioning the HbMPs on a grid and recording images by observing (i) transmitted electrons or secondary, low energy electrons emerging from the surface (SE2). The later are either (ii) detected by an Everhardt-Thomley-SE2-detector oriented laterally with respect to the incident electron beam or (iii) using an Inlens SE detector, sensitive against SE2 electrons emerging in a small solid angle symmetric to the backward direction. However, due to the properties of the biological particles none of the techniques is suited to obtain high contrast sharp images of the HbMP. Consequently, to record high contrast images, HbMPs were deposited on a plate and the sample was coated by sputtering platinum, resulting in a layer of 2 nm thickness. Besides contrast enhancement, charging is inhibited by the platinum coating. The SEM images shown in Figure 1b,c were recorded with the Inlens SE detector, which was operated at an acceleration voltage of 5 kV and a working distance of 5.2 mm.

AFM images were acquired with a NanoWizard 4 instrument (JPK BioAFM Business, Bruker Nano Surfaces, Berlin, Germany), mounted on a Zeiss inverted microscope. Since the HbMPs do not sufficiently adhere to glass surfaces, the microscopic slides were coated by a 50 µL drop of poly-L-ornithine (PLO). After incubation with PLO for 20 min the slides were washed twice with ultrapure water and blown dry with nitrogen flow. The HbMP suspension was prepared using a vortex mixer (5 min), a roll mixer (5 min) and an ultrasonic bath (5 min) to reduce agglomeration. Subsequently, the HbMP suspension was diluted 1:500 in acetated Ringer’s solution, 50 µL were pipetted on the coated slide and incubated in a humid chamber to allow particles to adhere. The AFM images were acquired with spatial resolution of 10 nm using a USC 0.3 cantilever, the calibration of which yielded a spring constant of 0.61 N m^−1^. In Figure 1d,e two HbMPs are shown with the height at 400 pN encoded in false colors. The long axis of the particle in Figure 1d is oriented parallel to the surface of the slide, while the particle in Figure 1e is fixated perpendicular to the surface of the slide. We observed that about 20% of the particles are oriented perpendicular. The electrostatic adhesion forces between the PLO coated slide and the particles immobilize the HbMPs with their respective orientation at the initial contact.

Both methods reveal the “peanut shape” of the HbMP with a long axis of approximately 800 nm and a waist of 400 nm. In addition, the surface exhibits fine structures in the range of 20–30 nm resulting from the MnCO_3_ salt template, which was dissolved in the final step when preparing the HbMPs.

### 3.4. Optical Setup

The optical setup for the measurement of collimated transmittance spectra is described by Gienger et al. [11] and is shown in Figure S1 of the Supplementary Materials. A high-power, continuous xenon light source (HPX-2000, Ocean Optics, Inc., Dunedin, FL, USA) irradiates the sample. Data were acquired in the spectral range between 200 nm and 1100 nm by a Maya2000 Pro spectrometer (Ocean Optics, Inc., Dunedin, FL, USA). In total, 7 Mirrors M1–M7 are used to provide a path length of approximately 1.5 m for the incident light beam and a distance of 1.5 m between the sample cuvette and the entrance aperture of the spectrometer (Figure S1). The lens L1 is used for collimation, i.e. to obtain an approximately parallel light beam. The apertures A1–A3 serve to reduce the divergence of the beam to about 0.01° (half angle), ensuring a plane-wave illumination from a single, well-defined direction. The samples are filled in a quartz cuvette (Hellma Analytics, Müllheim, Germany) with ℓ = (10 ± 0.01) mm optical path length. Aperture A4 blocks the light scattered in the non-forward direction by the sample. The spectrometer receives light from an observation angle as small as 0.02° (half angle). In contrast to a normal spectrophotometer, this serves to effectively suppress any light scattered at an angle to the incident beam. Hence, one can neglect unwanted contributions to the directed transmittance when analyzing the measurements. With the 50 µm entrance slit of the spectrometer, the spectral resolution is approximately 1.9 nm and spectra are sampled at about 0.45 nm per pixel on the CCD chip. Typically, spectra are generated within 10 s, thus the method allows rapid characterization of suspensions of HbMPs.

### 3.5. Protocol for Spectral Extinction Measurements

The HbMP stock suspension was pre-diluted 100-fold with water. The quartz cuvette with inner dimensions 10 mm × 10 mm was placed in the holder and filled with 2.2 mL of water. A dilution series was measured by subsequently pipetting volumes between 10 µL and 670 µL (in total) of the pre-diluted HbMP suspension into the cuvette, without touching it. This eliminates changes in reflections at the optical interfaces that would otherwise cause artifacts in the measured signals. Mixing of the fluids was achieved by pipetting back and forth into the cuvette several times. Six dilutions per sample were measured in this manner with transmittances ranging from *T* (300 nm) ≈ 92% and *T* (800 nm) > 99.5% for the lowest volume fraction (10 µL sample) down to *T* (300 nm) = 1.6% and *T* (800 nm) > 65% for the highest volume fraction (670 µL sample). The low-concentration high-transmittance measurements are prone to noise and measurement errors. On the other hand, the high-concentration measurements may show unwanted multiple-scattering effects. This was assessed by comparing the curves for Z(λ) or ${\bar{C}}_{\mathrm{ext}}\left(\lambda \right)$ obtained from the different dilutions. They agree well with each other as long as the transmittance is *T* (λ) ≥ 30%. That is to say, they differ by no more than an up- or downward shift on a logarithmic y-scale, i.e. by a re-scaling of the curve (due to volume errors of the pipette, compare Equations (1) and (2)). We used the highest concentration that shows no multiple-scattering effects for further analysis, due to the favorable signal-to-noise ratio. This corresponds to a sample volume of 130 µL and transmittances *T* (300 nm) = 30%, *T* (800 nm) > 89% for the curves in Figure 2 and Figure 3.

The extinction spectra of oxygenated/functional HbMPs and metHbMP were measured as described above. For deoxygenation of the HbMPs, argon gas was bubbled through the suspension to purge oxygen from the particles and liquid. The argon gas was fed into the cuvette through a metal capillary which remained in place during the recording of transmittance spectra. Foaming of the sample suspension did occur in this process, leading to a noticeable decrease in the particle concentration (or, equivalently to a reduced PPV) and hence to a systematic decrease in the determined extinction cross section. Since the VSECS is inversely proportional to the PPV, this error was corrected for by re-scaling the Z(λ) data of the deoxygenated HbMPs by 1.626 to match the data of oxygenated HbMPs, measured before bubbling, in a least-squares sense over the whole wavelength range between 300 to 800 nm. Non-rescaled spectra are provided in Figure S5 of the Supplementary Materials. The argon was applied for approximately 60 min and then turned off before recording the spectrum of the deoxygenated HbMPs. After deoxygenation, the sample was bubbled with air to re-oxygenate the particles within less than one minute.

### 3.6. Model for Optical Properties of Hemoglobin Microparticles

Complex RI data for the constituents of the HbMPs are required for the numerical simulation of extinction spectra. The light-scattering properties of a single spherical particle are determined by its diameter D and its complex refractive index (RI). For the light scattering simulations, the RI of the HbMPs was modelled by:

(5) $$n\left(\lambda \right)+i\kappa \left(\lambda \right)=n_{H_{2}O}\left(\lambda \right)+\;\beta_{\mathrm{Hb}}^{HbMP}\;\;\left[\alpha_{\mathrm{Hb}}\left(\lambda \right)+i\;\gamma_{\mathrm{Hb}}\left(\lambda \right)\right]+\;\;\beta_{\mathrm{Gl}/\mathrm{HSA}}^{HbMP}\;\left[\alpha_{\mathrm{HSA}}\left(\lambda \right)+i\;\gamma_{\mathrm{HSA}}\left(\lambda \right)\right]$$

which is the complex RI of an aqueous protein solution [11] containing a total hemoglobin mass concentration $\beta_{\mathrm{Hb}}$ and a heme-free globins (Gl)/HSA mixture at mass concentration $\beta_{\mathrm{Gl}/\mathrm{HSA}}$ [29,30]. The optical properties of HSA alone are used here to model both, HSA and (heme-free) globin. This approximation is justified, because the increments of the real and imaginary parts α(λ) and γ(λ) for globin are unknown but expected to be very similar due to the absence of a heme group and the similar molecular mass of the two globular proteins. The imaginary part of the respective RI increment, $\gamma_{y}\left(\lambda \right)$ with *y* = Hb, Gl/HSA, corresponds to the absorption spectrum of an aqueous protein solution. For hemoglobin, this imaginary RI increment contains contributions from the three hemoglobin components:

(6) $$\gamma_{\mathrm{Hb}}\left(\lambda \right)=\phi_{\mathrm{oxyHb}\;\;}\gamma_{\mathrm{oxyHb}}\left(\lambda \right)+\phi_{\mathrm{deoxyHb}\;\;}\gamma_{\mathrm{deoxyHb}}\left(\lambda \right)+\phi_{\mathrm{metHb}}\;\;\gamma_{\mathrm{metHb}}\left(\lambda \right)$$

where $\phi_{\mathrm{oxyHb}},\;\phi_{\mathrm{deoxyHb}}$ and $\phi_{\mathrm{metHb}}$ are the mass fractions of oxygenated, deoxygenated and methemoglobin, respectively. Similarly, the real RI increment of the hemoglobin component contains three contributions:

(7) $$\alpha_{\mathrm{Hb}}\left(\lambda \right)=\phi_{\mathrm{oxyHb}}\;\;\alpha_{\mathrm{oxyHb}}\left(\lambda \right)+\phi_{\mathrm{deoxyHb}\;\;}\alpha_{\mathrm{deoxyHb}}\left(\lambda \right)+\phi_{\mathrm{metHb}}\;\;\alpha_{\mathrm{metHb}}\left(\lambda \right)$$

**Figure 5:**
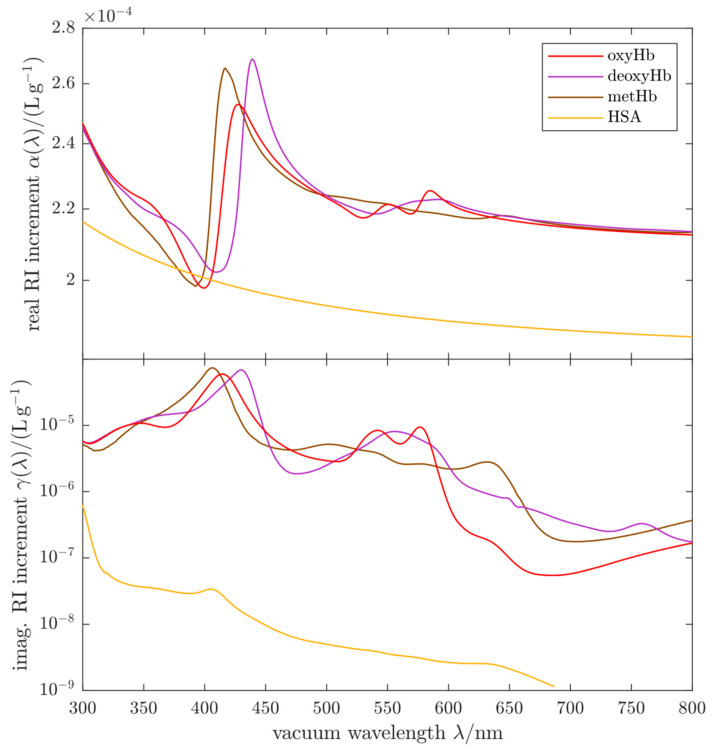
Complex refractive index increments of the Hb components and HSA used in our calculations of the extinction cross sections of HbMPs.

In Figure 5, the real and imaginary RI increments used in the simulations are shown. This enables us to simulate spectral extinction cross sections $C_{\mathrm{ext}}\left(\lambda \right)$ of single particles by Lorenz-Mie theory for any possible composition by changing $\beta_{\mathrm{Hb}}^{HbMP}\;$, $\beta_{\mathrm{Gl}/\mathrm{HSA}}^{HbMP}\;$, $\phi_{\mathrm{oxyHb}}$, $\phi_{\mathrm{deoxyHb}\;}$, $\phi_{\mathrm{metHb}}$ and thus n(λ)+iκ(λ) as well as the particle diameter D. Finally, VSECS spectra Z(λ) are obtained by integration over the particle size distribution (PSD).

### 3.7. Calculation of Extinction Cross Sections

The imaginary RI increments of the respective Hb components are linked to their molar extinction coefficients $\epsilon_{x}\left(\lambda \right)$ by:

(8) $$\gamma_{x}\left(\lambda \right)=\frac{\ln10}{4\;\pi }\;\frac{\epsilon_{x}\left(\lambda \right)}{4\;\;\;M{\left(\mathrm{Hb}\left(\mathrm{Fe}\right)\right)}^{}}\;\lambda$$

where x = oxyHb, deoxyHb or metHb and M(Hb(Fe)) is the molar mass of the hemoglobin monomer. An analogous relation holds true for $\gamma_{\mathrm{HSA}}\left(\lambda \right)$, the imaginary part of the RI increment of GI/HSA. In the simulations, we used the absorption spectra reported by Friebel and Meinke [31] for human oxyHb and deoxyHb and those reported by Zijlstra et al. [32]⁠ for human metHb. While such absorption spectra (i.e., γ(λ) data) are available for bovine Hb as used in the HbMPs, too, quantitative data for the real RI increment (i.e., α(λ) data) are only available for human Hb.

We use values for $\alpha_{\mathrm{oxyHb}}\left(\lambda \right)$ that were obtained from measurements of the extinction spectra of sphered oxygenated human erythrocytes [11]. For $\alpha_{\mathrm{deoxyHb}}\left(\lambda \right)$ and $\alpha_{\mathrm{metHb}}\left(\lambda \right)$ we use values that are consistent with the $\alpha_{\mathrm{oxyHb}}\left(\lambda \right)$ data and that were computed from the absorption spectra $\gamma_{\mathrm{deoxyHb}}\left(\lambda \right)$ and $\gamma_{\mathrm{metHb}}\left(\lambda \right)$ for human hemoglobin using Kramers-Kronig relations, i.e. a fundamental causality principle in physics [18,31]. The absorption spectra of human and bovine hemoglobin are known to differ very little [32] and consequently the real parts of the RI differ very little, too. Hence the error made by assuming the optical properties of human Hb even though the HbMPs contain bovine Hb is small. The values for $\alpha_{\mathrm{HSA}}\left(\lambda \right)$, used to model the globin/HSA mixture in the HbMPs, were determined from extinction spectroscopy with well-characterized quasi-monodisperse polystyrene beads suspended in the HSA solution. The $\gamma_{\mathrm{HSA}}\left(\lambda \right)$ spectra were obtained by standard spectrophotometry. The details of the RI determination of HSA are described in the corresponding section of the Supplementary Materials.

To compute the complex RI of the HbMPs for light scattering simulations, the intra-particle protein concentrations were set to $\beta_{\mathrm{Hb}}=120{g\;L}^{-1}$ and $\beta_{\mathrm{Gl}/\mathrm{HSA}}=130{g\;L}^{-1}$, corresponding to the results of the density and PPV measurements. For our analysis we use the RI of pure water [33] for the host medium surrounding the particles in accordance with the measurement conditions, i.e. the HbMP suspension was diluted in pure water. The increase of the RI due to dissolved proteins from the storage solution of the HbMPs is negligible due to the high dilution (>1000-fold) of the sample in the extinction measurements.

In general, the dependence of $C_{\mathrm{ext}}\left(\lambda \right)$ for a single particle on both, the complex RI n(λ)+iκ(λ) and the diameter D is highly nonlinear, thus requiring to repeat the computations for every composition and size. For a given particle size distribution (PSD), labeled by the symbol p(D), the ensemble-averages are computed as:

(9) $$\;{\bar{C}}_{\mathrm{ext}}\left(\lambda \right)=\int_{D_{\min}}^{D_{\max}}C_{\mathrm{ext}}\left(\lambda ;D\right)\;\;p\left(D\right)\;dD\;\;\;\wedge \;\;\;\bar{V}=\int_{D_{\min}}^{D_{\max}}\;\frac{\pi }{6}\;D^{3}\;p\left(D\right)\;dD$$

to obtain $Z\left(\lambda \right)={\bar{C}}_{\mathrm{ext}}\left(\lambda \right)/\bar{V}$. The PSD is assumed to be a log-normal distribution:

(10) $$p\left(D\right)=\frac{1}{\sqrt{2\pi }\;\sigma_{D}}\;\frac{1}{D}\;\exp\left[\frac{-\ln{\left(D/\mu_{D}\right)}^{2}}{2\sigma_{D}^{2}}\right]$$

which fits the DLS and AC results well. The parameter $\mu_{D}$ coincides with the median diameter and $\sigma_{D}$ is a measure for the relative distribution width. Note that the distribution width w(D) defined for the measured PSDs correlates with but is not identical to the parameter *σ_D_*. Using the quantiles *Q* of the PSD, which can be expressed in terms of *µ_D_* and *σ_D_*, the distribution width is given as w(D)= Q(84%) − Q(16%). For the simulations, we used those parameter values that yield the same median and absolute distribution width w(D) as the DLS measurements, i.e. $\mu_{D}=760\;µm$ and $\sigma_{D}=25.8\%$. Simulations using the AC distribution parameters, i.e. $\mu_{D}=996\;µm$ and $\sigma_{D}=15.1\%$ as well as other sets of parameters are provided in Figures S2 and S3 of the Supplementary Materials. Ideally the integration limits used with a log-normal PSD would be $D_{\min}=0,\;D_{\max}=\infty$. In practice, we used numerical integration with bounds $D_{\min}\leq Q\left(0.1\%\right),\;D_{\max}\geq Q\left(99.9\%\right)$ and a step width ≤20 nm.

### 3.8. Soft X-ray Fluorescence

The NEXAFS experiments were carried out at the plane grating monochromator (PGM) beamline [34] for undulator radiation in the PTB laboratory of the BESSY II electron storage ring in Berlin (Germany). This beamline provides soft X-ray radiation of high photon flux and spectral purity in the photon energy range of 78 eV–1860 V (15.7−0.659 nm) and allows for dedicated experiments on light elements such as carbon, nitrogen, or oxygen. A UHV chamber [35], which is equipped with a scanning table for sample alignment, photo diodes for normalization and a silicon drift detector (SDD) for detection of the emitted fluorescence radiation from the sample. The SDD detector is equipped with a thin Be-window in order to improve the ratio between the detected Fe-L and the intense oxygen (O-Kα) fluorescence radiation originating from the water-based solution containing the HbMPs. The liquid must be transferred into the UHV in order to avoid the strong attenuation of soft x-rays in all kinds of media. In this work, the hemoglobin microparticle suspensions are probed using a liquid sample cell with an ultra-thin entrance window (thickness 100 nm) made from Si_3_N_4_ as an appropriate separator between liquid and vacuum [36]. The liquid cell was directly mounted into the UHV chamber and has a total sample volume of about 0.4 mL. The actual interaction volume, defined by the excitation beam and the solid angle of observation (cylindrical with diameter 4 mm and depth 3 mm) behind the window amounts to about 40 µL.

In Figure 4a, two X-ray fluorescence spectra of HbMP suspensions (dot-dashed red and dashed brown traces) are shown, recorded by scanning the excitation energies between 698 to 736 eV (1.776−1.685 nm). The HbMP stock solution was enriched to about a PPV of 60% to ensure that the exciting X-ray beam is absorbed by the HbMPs. The spectra shown are averaged over three repeat measurements each, conducted on the same sample to prove that influences due to sedimentation, irradiation induced effects or sample degradation were not relevant. Besides the spectra for oxyHbMP (dot-dashed red line) and the metHbMP (dashed brown trace), we include absorbance reference spectra [18] for Fe(II) and Fe(III) as blue and green lines.

### 3.9. Packed Particle Volume

Centrifugation with a standard hematocrit centrifuge (Hettich EBA 12, Andreas Hettich GmbH, Tuttlingen, Germany) was used to determine the solid fraction or packed particle volume (PPV), i.e. the ratio of the volume occupied by particles to the total volume in the suspension of hemoglobin microparticles. The suspension of HbMPs was filled into 10 BRAND^®^ micro hematocrit capillary (BRAND GmbH + Co KG, Wertheim, Germany), typically the height amounts to 55 mm. The protocol usually applied to determine the hematocrit value in whole blood samples, described in the standard DIN 58933-1 [37], had to be modified since sedimentation of HbMPs was observed to be incomplete. To ensure that the end point is reached, the relative centrifugation force was increased from 5000 to 6250 g, g = 9.8 m s^−2^ being the terrestrial gravitational acceleration, and the centrifugation time was increased to 21 min compared to 16 min. For readout of the hematocrit tubes, an electronic scale (Peak Optics, La Quinta, CA, USA) with a 100 µm resolution was used. We observed that the HbMPs are flexible and the corresponding compression results in a lower PPV if measured directly after centrifugation. To account for this effect, we measured the PPV of the 10 capillaries as a function of time and determined the mean value and standard deviation. To avoid systematic deviations, three persons were involved, characterized by different symbols (circle, diamond, cross) in Figure S4 of the Supplementary Materials. The data were modeled by a dose response function yielding a value of PPV = 0.1992(3) (see Table 1), the expansion of the PPV amounts to 0.0084 corresponding to about 4%.

### 3.10. Dynamic Light Scatter

Particle size was determined by dynamic light scattering (DLS) employing a Litesizer^TM^ 500 (Anton Paar GmbH, Graz, Austria) instrument. To prove that the instrument was operated in its specified transmittance range a series of six dilutions was prepared for DLS measurements covering particle concentrations 50 × 10^3^ nL^−1^ ≥ *C* ≥ 103 nL^−1^. The resulting transmittance values range from 0.01 to 60%. For suspensions with low particle concentrations the Litesizer^TM^ was operated in the side scatter mode (red symbols in Figure S6) while for high particle concentrations backscatter was measured (blue symbols in Figure S6). The result, i.e. the median diameter of the intensity weighted distribution, is listed in Table 1. For consistency the median value is given, since we analyzed our extinction measurements taking the median of the size distribution. The uncertainty $u\left(D_{median}\right)$ was estimated accounting for the standard deviation of the six measurements of the dilution series. It should be noted that the particle size distributions, derived from the measured temporal autocorrelation function, can be described to a good approximation by logarithmic normal distributions. Results for such fits agree well with the averaged value for the width w(D) (Table 1) obtained by numerical analysis of the distributions for the 16% to 84% summarized intensities.

### 3.11. Analytical Centrifugation

Besides DLS, we applied analytical centrifugation (AC) to derive the particle size distribution, characterized by median and width. The type of centrifugation used is based on cuvettes containing the diluted samples and transmission measurements and generally abbreviated as cAC-turb [38]. For simplification, we use the abbreviation AC in this paper. Two different suspensions were prepared from the HbMP stock suspension by adding acetated Ringer’s solution (Serumwerk Bernburg AG, Bernburg, Germany) to obtain dilutions of 1:40 and 1:75. These dilutions were selected to meet the requirements of the LUMiSizer centrifuge (LUM GmbH, Berlin, Germany), i.e. the initial transmittance should be between 30% and 50%. Each suspension was pipetted to 3 disposable cuvettes of 2 mm path length. The centrifugation force was increased in several steps to cover a large size range from 100 nm to several micrometer. Total measurement time amounted to about 18 min. Since for the 1:40 dilution the initial transmittance amounted to 40% and for the 1:75 dilution to 55%, the median diameter given in Table 1 is determined from the measurements of the 1:40 dilution only. As observed with DLS, the particle size distributions measured by AC, derived from the time dependence of the interface between the suspension and the transparent supernatant, is well described by logarithmic normal distributions. To estimate the uncertainty contribution due to the uncertainty of the density, the AC measurements were analyzed using different particle densities and the solvent’s density, i.e. acetated Ringer’s solution. In Figure S7, we plotted the result for the median sphere equivalent particle diameter versus the particle density in the vicinity of the values derived by the density measurements. Hence, the uncertainty in Table 1 for the sphere equivalent diameter determined by AC accounts for the standard deviation from the results obtained for the 3 cuvettes of the 1:40 dilution and the uncertainty of the density measurements.

### 3.12. Concentration of Hemoglobin Microparticles

The concentration of HbMPs in the stock suspension was measured directly by flow cytometry and calculated according to:

(11) $$C_{0}=\frac{\mathrm{PPV}}{\left(4/3\right)\;\;\pi \;\;{\left(D_{median}^{}/2\right)}^{3}}$$

taking into account the median particle diameters derived by DLS and AC. Flow cytometric measurements were performed with a CyFlow Cube 8 instrument (Sysmex Partec, Görlitz, Germany), configured with a 50 mW 488 nm solid state laser and observation channels for forward light scatter (FSC), side scatter (SSC) and three fluorescence channels. This system offers volumetric counting by start stop electrodes and a computer controlled pump for high accuracy syringes. The stock solution of the HbMPs was diluted by about a factor 1:106 to adjust the count rate in the range between 1 kHz and 5 kHz. Whereas autofluorescence of HbMPs could not be detected, in the FSC versus SSC diagram HbMPs (Figure S8a) can be discerned from the background and the debris. To assess the sensitivity, we include a scatter diagram of a mixture of polystyrene microspheres with diameters of 220 nm and 500 nm (Figure S8b). The clusters of these two populations are clearly visible, additional populations at higher signal intensities are caused by particle agglomerates. The events localized close to the trigger level are due to electronic noise or contaminating particles in the sheath fluid. Gating the cluster representing the HbMPs in the FSC versus SSC dot plot, we obtain the pulse height distribution of the SSC intensities, depicted in the histogram in Figure S8c. The same gate was used for the polystyrene particles, the corresponding histogram is included in Figure S3c for comparison. It is evident that the maximum of the HbMP distribution is located at lower side scatter intensity compared to the 220 nm polystyrene particles, although their sphere equivalent diameter is approximately 4 times and their volume 40 times larger. The difference in light scattering intensity is caused by the different polarizability of the particles, i.e. their refractive indices, being 1.605 for polystyrene and (1.390 + 3.6 × 10^−4^ i) for the oxyHbMP at 488 nm. Hence, for flow cytometric detection of HbMPs the sensitivity of the flow cytometer should be sufficient to detect 100 nm polystyrene micro spheres. The measurement time amounted to 30 s, 44,000 HbMPs out of 70,000 total events were delineated by setting the gate as illustrated in Figure S8a. Taking into account the volume fraction (7.5 × 10^−7^) of the HbMP suspension in the measurements sample and the measurement volume (200 µL), we obtain the concentration of HbMPs given in Table 1, i.e. (293 ± 30) pL^−1^. Compared to the values derived from the measurements of the particles’ volume by DLS and AC this value is the smallest, possibly caused by adhesion loss on container and tube walls or agglomerates of HbMPs, detected as single event. However, because of the relatively large range for the concentrations measured with different methods, as described in the Section Physical Properties of HbMPs, we introduce the quantity Z(λ), independent on the concentration, to analyze the extinction spectra. Hence, uncertainties due to the concentration are avoided.

### 3.13. Density of Hemoglobin Microparticles

The densities of the stock suspension of HbMPs, the supernatant and reagents involved in the various preparations were measured by a mechanical oscillator device [28]. We used a prototype model µDMA (Hans Stabinger GmbH, Graz, Austria) which was developed to allow density determination for sample volumes of typically 100 µL with an accuracy of about 10^−4^ g mL^−1^. The densities of the HbMP suspension and the various reagents in Table 1 are directly determined with the µDMA instrument from up to 10 repeated measurements. As uncertainty, the standard deviation is reported. The density of water was measured to validate the calibration of the instrument. As already stated in the Results and Discussion (Section 2.5. Physical properties of HbMPs), the density of the HbMPs is calculated from the PPV and the densities of the HbMP suspension and the supernatant according to:

(12) $$\rho_{}^{HbMP}=\frac{1}{\mathrm{PPV}}\;\left(\;\rho_{}^{sus}-\;\;\;\rho_{}^{sup}\;\right)+\rho_{}^{sup}$$

This procedure was applied to determine the densities of erythrocytes as function of the mean corpuscular hemoglobin concentration (MCHC) [21]⁠. The high accuracy density determination of the HbMPs allows to model the composition of the HbMPs, since besides Hb, heme-free globin (Gl) and human serum albumin (HSA) are incorporated in the particles, as discussed in the Results section. In this context, we use the values in Table 1 for the density increments $DI_{\mathrm{Hb}}^{}$ of bovine hemoglobin and $DI_{\mathrm{Gl}/\mathrm{HSA}}^{}$ and of a 1:1 mixture of Hb (in lack of a value for heme-free globin) and HSA. The density increment of a solution in a given solvent is defined as $DI_{\mathrm{solute}}^{}=\;\left(\;\partial \rho_{\mathrm{solution}}^{}/\partial \beta_{\mathrm{solute}}^{}\right)$. A higher uncertainty is assumed for $DI_{\mathrm{Gl}/\mathrm{HSA}}^{}$ to account for the possibility of Gl to HSA ratios other than 1:1.

### 3.14. Total Hemoglobin Concentration

The total Hb concentration in the suspension of HbMPs was determined by spectrophotometry using the AHD method [26,27], which was demonstrated to allow high accuracy reference measurements of the total hemoglobin concentration in whole blood samples by comparison with the hemiglobincyanide (HiCN) procedure [39,40]. An aliquot of the HbMP stock suspension was vortexed for 10 min for homogenization. Due to the cross-linking of the hemoglobin and albumin molecules, an enzymatic digestion is required to completely degrade the HbMP. For this purpose, a Pronase solution with a concentration of about 10 mg mL^−1^ was used. A mixture containing Pronase solution and HbMP suspension with a 1:1 mass ratio was incubated for 30 min at 50 °C. The resulting Hb solution was used to prepare a dilution series with volume fractions between 0.116 and 0.215 for absorption measurements using a Cary 5000i spectrophotometer (Agilent Technologies, Waldbronn, Germany). A second “reference” solution was prepared just containing Pronase with the same concentration as in the Hb-solution. A corresponding dilution series was prepared, filled in an identical quartz cuvette, which was positioned in the reference beam of the spectrophotometer. Hence, the absorption spectra (Figure S9) represent the contribution caused by the digested HbMPs. From the spectra, the spectral absorbance A(λ) at 574 nm is determined for the different dilutions $\varphi_{i}$ and the mass concentration is calculated according to:

(13) $$\beta_{\mathrm{Hb}}^{sus}=\frac{A\left(\lambda \right)\;M\left(\mathrm{Hb}\left(\mathrm{Fe}\right)\right)}{\ell \;\;\varepsilon \left(\lambda \right)\;\;\varphi_{i}}$$

The molar mass M(Hb(Fe)) of the monomeric Hb(Fe) amounts to 16114.5 g mol^−1^, the absorption lengths of the quartz cuvettes were d = 10 mm and the molar extinction coefficient is given by ε (λ = 574 nm) = 6945 L mol^−1^ cm^−1^. Finally, the total Hb concentration (Table 2a) is determined as the weighted average of the different dilutions. The uncertainty given in the table is estimated by repeating the procedure several times at various days.

### 3.15. Hemoglobin Concentration in Hemoglobin Microparticles

To determine the content of hemoglobin as well as heme-free globin and HSA, we calculate the density difference between the HbMP suspension and acetated Ringer’s solution according to:

(14) $$\rho_{}^{sus}-\rho_{}^{RAc}={\mathrm{DI}}_{\mathrm{Hb}}^{}\;\;\beta_{\mathrm{Hb}}^{sus}+{\mathrm{DI}}_{\mathrm{Gl}/\mathrm{HSA}}^{}\;\;\beta_{\mathrm{Gl}/\mathrm{HSA}}^{sus}$$

Each of these two components contributes according to its mass concentration β and its density increment given in Table 1 and Table 2. For aqueous protein solutions it was found that the density increments are almost constant over a wide range of concentrations [41]⁠. The values in Table 1 were taken from this Reference and converted from water or phosphate buffer to RAc and extrapolated to 23 °C. Rearrangement of Equation (14) allows the calculation of the concentration of heme-free globin/HSA in the suspension by:

(15) $$\beta_{\mathrm{Gl}/\mathrm{HSA}}^{sus}=\frac{\rho_{}^{sus}-\;\rho_{}^{RAc}-{\;\mathrm{DI}}_{\mathrm{Hb}}\;\beta_{\mathrm{Hb}}^{sus}}{{\mathrm{DI}}_{\mathrm{Gl}/\mathrm{HSA}}^{}}$$

The resulting concentration $\beta_{\mathrm{Gl}/\mathrm{HSA}}^{sus}$ = 27.7 g L^−1^ (Table 2a) corresponds to a ratio between non-heme proteins and hemoglobin or relative concentration of $\left(\beta_{\mathrm{Gl}/\mathrm{HSA}}^{sus}/\beta_{\mathrm{Hb}}^{sus}\right)$ = 1.0868. In the production process of the HbMPs, only a small fraction of the HSA is used for coating or in washings steps and the majority of HSA is co-precipitated and cross-linked together with the Hb. Hence, we will assume that the HSA to Hb ratio is the same in the supernatant as in the particles. This means that the mass concentration of hemoglobin in the supernatant $\beta_{\mathrm{Hb}}^{}$ follows in analogy to Equation (14) from the density difference between the supernatant and Ringer’s solution:

(16) $$\rho_{}^{sup}-\rho_{}^{RAc}={\mathrm{DI}}_{\mathrm{Hb}}^{}\;\;\beta_{\mathrm{Hb}}^{sup}+\;{\mathrm{DI}}_{\mathrm{Gl}/\mathrm{HSA}}^{}\;\;\beta_{\mathrm{Gl}/\mathrm{HSA}}^{sup}$$

Replacing the mass concentration of the Gl/HSA proteins $\beta_{\mathrm{Gl}/\mathrm{HSA}}^{}$ by the product of the ratio in the suspension and the mass concentration $\beta_{\mathrm{Hb}}^{sup}$ of Hb in the supernatant and rearranging Equation (16) the mass concentration of Hb in the supernatant is given by:

(17) βHbsup=( ρsup−ρRAc )DIHb  +  DIGl/HSA  (βGl/HSAsusβHbsus)

Finally, the mass concentration of Hb proteins $\beta_{\mathrm{Hb}}^{HbMP}$ in the HbMP is calculated taking into account that the total Hb concentration in the suspension is the sum of the concentrations in the HbMP according to

(18) $$\beta_{\mathrm{Hb}}^{sus}=\mathrm{PPV}\;\;\beta_{\mathrm{Hb}}^{HbMP}+\left(1-\mathrm{PPV}\right)\;\beta_{\mathrm{Hb}}^{sup}$$

with the contributions weighted by the packed volume fraction PPV = 0.1992(3). Hence, the mass concentration of Hb in the HbMPs follows as

(19) $$\beta_{\mathrm{Hb}}^{HbMP}=\frac{1}{\mathrm{PPV}}\;\left\{\;\beta_{\mathrm{Hb}}^{sus}-\left(1-\mathrm{PPV}\right)\;\beta_{\mathrm{Hb}}^{sup}\;\right\}$$

The results for these quantities are summarized in Table 2a.

### 3.16. Oxygen Release Measurements

To determine the mass concentration of functional, i.e. oxyhemoglobin in HbMPs, we measured the ferricyanide-induced release of hemoglobin-bound oxygen into the surrounding medium [23,24]. The dissolved oxygen of the three different HbMP suspensions (oxyHbMP, deoxyHbMP, metHbMP) was measured by a miniaturized optical needle type oxygen sensor (oxygen microsensor NTH-PSt7, PreSens–Precision Sensing GmbH, Regensburg, Germany) connected to an oxygen meter with data logging (Microx 4, PreSens—Precision Sensing GmbH). For saturation with oxygen, 1 mL of HbMP suspensions and hemoglobin solutions were left for equilibration for 10 min under stirring. Thereafter, 50 µL to 100 µL of 10% ferricyanide (K_3_[Fe(CN)_6_]) were added to detect the concentration change of dissolved oxygen with an acquisition rate of one data point per second. When a stable value was reached, the measurement was stopped. Further adding of ferricyanide did not lead to a pO_2_ increase. While there was no increase of dissolved oxygen when ferricyanide was added to the control (aqua dest.), a hemoglobin concentration dependent change in pO_2_ was observed. Taking into account the difference of final and initial pO_2_ and assuming that all released oxygen was previously bound to hemoglobin, a standard curve for the hemoglobin concentration versus change of pO_2_ was generated and used to derive the mass concentration of the functional hemoglobin in the HbMPs. Apart from the different preparation to generate deoxyHb and metHb (see 3.1. Experimental Designs), the same protocol was applied to determine the dissolved oxygen in suspensions of deoxyHbMP and metHbMP.

## 4. Conclusions

We have demonstrated that spectral optical extinction measurements of hemoglobin microparticles and analysis (SEMPA) is a valuable tool to monitor and optimize the production of microparticles and sub-micrometer particles and to evaluate the effect of relevant influencing factors. Extinction data were analyzed by Lorenz-Mie theory to yield the relative concentrations of metHb, oxyHb and deoxyHb and thus the functionality of microparticle based artificial blood substitutes. In particular, by ensuring that multiple scattering events are negligible, the—ensemble averaged—particle properties are easily accessible. This is a significant advantage with respect to the oxygen release measurements as the usually applied technique, which cannot distinguish between Hb inside and outside the microparticles. We applied the SEMPA technique to specify hemoglobin microparticles, fabricated by the co-precipitation—cross-linking—dissolution technique. For validation of the determined ratio of functional Hb/metHb, we applied NEXAFS fluorescence measurements as complementary method and observed good agreement between these results. The observed spectral extinction cross sections cannot be described by calculations assuming that the HbMPs are composed of the three component oxyHb, deoxyHb and metHb solely. Our results prove that a considerable portion of heme-free globin/HSA of about 50% of the total protein is present in the investigated particles, a value which was confirmed by high accuracy density measurements.

The SEMPA method could potentially replace the elaborate oxygen release analysis by adding a flow-through system. Integration of a gas exchanger would allow the reversible oxygenation and deoxygenation of the sample including kinetic studies.

## 5. Patents

On behalf of J.G. and J.N. the Physikalisch-Technische Bundesanstalt has filed a patent application (DE 10 2017 121 587 A1) describing the optical procedure for the simultaneous determination of particle properties and particle measuring device. Issues related to intellectual properties will be managed by the Innovation and Technology Transfer Department at the Physikalisch-Technische Bundesanstalt.

## Figures and Tables

**Table 1:** Summary of results for the characterization of the HbMP suspension. Besides the (sphere equivalent) median particle diameter and the particle distribution width, the packed particle volume, the concentration and densities relevant for our analysis are listed.

Quantity	Symbol	Value		Uncertainty	Unit	Relative Uncertainty		Measurement Method
Median diameter (Intensity Weighted Distribution)	*D_median_ =*	760	±	54	nm	7.2	%	DLS
Distribution Width (16–84%)	*w*(*D*) *=*	395	±	94	nm	23.8	%	
Relative Distribution Width	*w_rel_*(*D*) *=*	52	±	12	%	23.8	%	
Median Diameter (Intensity Weighted Distribution)	*D_median_ =*	996	±	26	nm	2.6	%	Analytical Centrifugation (AC)
Distribution Width (16–84%)	*w*(*D*) *=*	300	±	20	nm	6.7	%	
Relative Distribution Width	*w_rel_*(*D*) *=*	30	±	2	%	6.7	%	
Packed Particle Volume of HbMP Stock Suspension	PPV =	0.1992	±	0.0003				Centrifugation
Particle Concentration of HbMP Stock Suspension	*C_0_ =*	866	+	259	pL^−1^			PPV, Particle Volume Determined by DLS
			−	162				
	*C_0_ =*	385	+	190	pL^−1^			PPV, Particle Volume Determined by AC
			−	180				
	*C_0_ =*	293	±	30	pL^−1^			Flow Cytometry
Densities *ρ* at 23 °C								
HbMP Stock Suspension	*ρ^sus^* =	1.01639	±	0.00010	g mL^−1^			Mechanical Oscillator Device
Supernatant of HbMP Stock Suspension	*ρ^sup^* =	1.00422	±	0.00010	g mL^−1^			
Acetated Ringer’s Solution	*ρ^RAc^* =	1.00320	±	0.00010	g mL^−1^			
Pronase Solution	*ρ^Pro^* =	1.00101	±	0.00009	g mL^−1^			
H_2_O	*ρ^water^* =	0.99749	±	0.00003	g mL^−1^			
HbMP	*ρ^HbMP^* =	1.0653	±	0.0006	g mL^−1^			Mechanical Oscillator Device, PPV
Density Increment Gl/HSA	DI _Gl/HSA_ =	0.2505	±	0.0056				Literature Value
Density Increment Hb	DI _Hb_ =	0.2450	±	0.0010				Bovine Hb, Literature Value

**Table 2:** (a) Total hemoglobin concentrations in the various components of the HbMP suspension and total concentration of heme-free globin/HSA. (b) Concentrations of functional Hb and relative concentrations of metHb. * The HbMP suspension with all Hb variants converted to metHb was used for validation of the spectral extinction measurements and the X-ray fluorescence by setting the value to 100%.

a	Mass Concentrations			
	Total Hb in Stock Suspension	Hb in Supernatant of Stock Suspension	Heme-Free Globin/HSA in Stock Suspension	HbMP
Results for PPV = 19.9%	$\beta_{\mathrm{Hb}}^{sus}$	$\beta_{\mathrm{Hb}}^{sup}$	$\beta_{\mathrm{Gl}/\mathrm{HSA}}^{sus}$	$\beta_{\mathrm{Hb}}^{HbMP}$
Method	Enzymatic Digestion, AHD Conversion and Spectrophotometry	Results for Total Hb, Density Measurements by Mechanical Oscillator Device and PPV		
Sample Preparation	g L^−1^	g L^−1^	g L^−1^	g L^−1^
oxyHbMP, deoxyHbMP, metHbMP	25.5 ± 0.5	2.0 ± 0.2	27.7 ± 0.9	120.1 ± 2.7
**b**	Mass Concentration of Functional Hb	Relative Concentration of Non-functional Hb/metHb		
Results for PPV = 19.9%				
Method	Oxygen Release	Oxygen Release	Spectral Extinction Measurements	X-ray Fluorescence
Sample Preparation	g L^−1^	%	%	%
oxyHbMP	11.8 ± 0.7	54 ± 3	35 ± 5	35 ± 5
deoxyHbMP	4.8 ± 0.9	81 ± 3	**−**	**−**
metHbMP	2.8 ± 0.4	89 ± 1	100 *	100 *

− value not determined by Spectral Extinction Measurements, X-ray Fluorescence not sensitive to deoxyHb.

## Data Availability

All data is contained within the article or supplementary material as Figure or Table. The numerical data represented in the Figures are available on request from the corresponding author.

## Supplementary Materials

The following are available online at https://www.mdpi.com/1422-0067/22/4/1753/s1, List of abbreviations; List of symbols; Figure S1: Optical Setup for collimated transmittance measurements; Figure S2: Influence of median particle size on the volume-specific extinction cross section for HbMPs of given relative size distribution width $\sigma_{D}^{}$ = 25.8% and composition; Figure S3: Influence of distribution width parameter $\sigma_{D}$ of the log-normal particle size distribution on the volume-specific extinction cross section of HbMPs at fixed median *D_median_* = 760 nm; Figure S4: Packed particle volume (PPV) or solid fraction of the HbMP stock suspension as function of time; Figure S5: Oxygenation—deoxygenation and effect of particle loss; Paragraph “RI determination of HSA”; Figure S6: Particle diameter of HbMP measured by DLS for different concentrations; Figure S7: Correlation of the results for the particle size determined from sedimentation measurements and the density of the HbMP; Figure S8. Flow cytometric measurements of HbMP and polystyrene microspheres; Figure S9: Absorption spectra of the HbMP suspension resulting from different dilutions.
