# Supplementary material for: Function of Hemoglobin-Based Oxygen Carriers: Determination of Methemoglobin Content by Spectral Extinction Measurements
Source: Int J Mol Sci. 2021 Feb 10;22(4):1753. doi: 10.3390/ijms22041753 (PMC7916497; doi:10.3390/ijms22041753)
Supplement: Supplementary file 1 [file ijms-22-01753-s001.pdf]

SUPPLEMENTARY INFORMATION

Determination of methemoglobin in hemoglobin based oxygen carriers by spectral extinction measurements

Kathrin Smuda[a,b*], *Jonas Gienger*[b], *Philipp Hönicke*[b], *Jörg Neukammer*[b],

[a]Institute of Transfusion Medicine, Charité - Universitätsmedizin Berlin, Germany;

[b]Physikalisch-Technische Bundesanstalt, Berlin, Germany

Abbreviations

AC	Analytical centrifugation
AHD	Alkaline hematin and detergent method for total Hb determination
AFM	Atomic force microscopy
CCD	Charge coupled device
deoxyHb	Deoxygenated variant of hemoglobin
deoxyHbMP	Suspension of HbMPs prepared to deoxyHb
DLS	Dynamic light scattering
DCHb	Diaspirin cross-linked hemoglobin
EDTA	Ethylenediaminetetraacetic acid
FCM	Fow cytometric measurement
Gl	Heme-free globin
Hb	Hemoglobin
HbMP(s)	Hemoglobin microparticle(s)
HBOC	Hemoglobin based oxygen carrier
HiCN	Cyanhemoglobin
HSA	Humans serum albumin
metHb	Methemoglobin with Fe in the ferric (Fe(III)) state
metHbMP	Suspension of HbMPs treated to convert Hb into metHb
NEXAFS	Near edge X-ray absorption fine structure
oxyHb	Oxygenated variant of hemoglobin
oxyHbMP	Suspension of HbMPs prepared to obtain oyxHb, i.e.oxygen saturation
PGM	Plane grating monochromator
PLO	Poly-L-Ornithine

PSD	Particle size distribution
RI	Refractive Index
SDD	Silicon drift detector
SEM	Scanning electron microscopy
SE2	Low energy secondary electrons in SEM experiments emerging from the sample surface
SEMPA	Spectral extinction measurement in particle suspensiona and analysis
VSECS	Volume specific extinction cross section

Symbols

$\alpha_{\mathrm{Hb}}(\lambda)$	Increment of the real part of the RI as function of the total Hb concentration
$\alpha_{\mathrm{HSA}}(\lambda)$	Increment of the real part of the RI as function of the concentration of HSA
$\alpha_{\mathrm{x}}(\lambda)$	Increment of the real part of the RI as function of the concentration of the Hb variants $x \in$ { oxyHb, deoxyHb, metHb }
$\beta_{\mathrm{Gl/HSA}}^{HbMP}$	Mass concentration of Gl/HSA in the HbMPs
$\beta_{\mathrm{Hb}}^{HbMP}$	Mass concentration of Hb in the HbMPs
$\beta_{\mathrm{Gl/HSA}}^{sup}$	Mass concentration of Gl / HSA in the supernatant of the HbMP stock suspension
$\beta_{\mathrm{Hb}}^{sup}$	Mass concentration of Hb in the supernatant of the HbMP stock suspension
$\beta_{\mathrm{Gl/HSA}}^{sus}$	Mass concentration of Gl / HSA in the HbMP stock suspension
$\beta_{\mathrm{Hb}}^{sus}$	Mass concentration of Hb in the HbMP stock suspension
C_0	Concentration of HbMPs in the stock suspension
$C_{ext}(\lambda)$	Spectral extinction cross section
$\overline{C}_{ext}(\lambda)$	Ensemble averaged spectral extinction cross section
D	Diameter of HbMPs
D_{median}	Median diameter of the PSD of HbMPs

DI_{Hb}	Density increment of Hb
$DI_{Gl/HSA}$	Density increment of Gl/HSA
$\epsilon_x(\lambda)$	Molar extinction cross sections of the Hb variants $x \in \{$ oxyHb, deoxyHb, metHb $\}$
φ	Volume fraction of stock suspension in the measurement suspension
ϕ_x	Mass fractions of the Hb variants $x \in \{$ oxyHb, deoxyHb, metHb $\}$
$\gamma_{\mathrm{Hb}}(\lambda)$	Increment of the imaginary part of the RI as function of the total Hb concentration
$\gamma_{\mathrm{HSA}}(\lambda)$	Increment of the imaginary part of the RI as function of the HSA concentration
$\gamma_{\mathrm{x}}(\lambda)$	Increment of the imaginary part of the RI as function of the concentration of the Hb variants $x \in \{$ oxyHb, deoxyHb, metHb $\}$
$\kappa(\lambda)$	Imaginary part of the RI
ℓ	Absorbance length of the cuvette
λ	Wavelength
M_{Hb}	Molar mass of the hemoglobin tetramer
$n(\lambda)$	Real part of the RI
PPV	Packed particle volume
ρ^{HbMP}	Density of HbMPs
ρ^{Pro}	Density of Pronase solution (0.9982g in 100.2617g H_2O)
ρ^{RAc}	Density of acetated Ringer's solution
ρ^{sup}	Density of supernatant of HbMP stock suspension
ρ^{sus}	Density of HbMP stock suspension
$T(\lambda)$	Spectral transmittance
$\overline{T}(\lambda)$	Ensemble averaged spectral transmittance
$\overline{V}$	Median volume of the HbMPs
$Z(\lambda)$	Volume specific extinction cross section

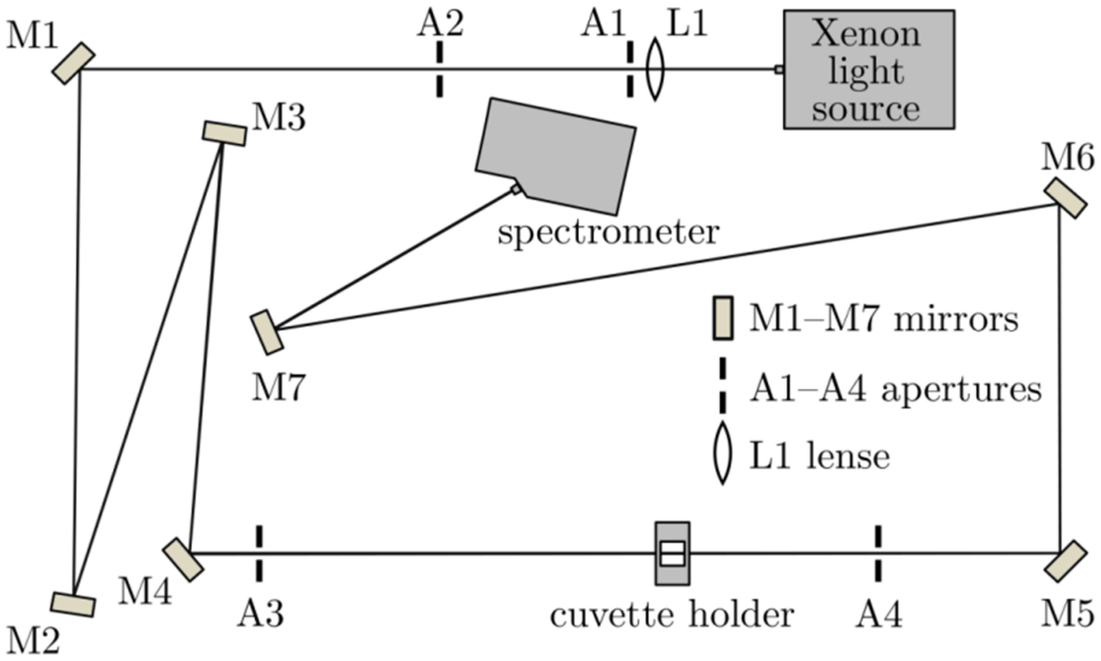

Figure S1. Optical Setup for collimated transmittance measurements. Mirrors to increase the length of light path as well as the apertures ensure a low divergence of both the incident and detected beams.

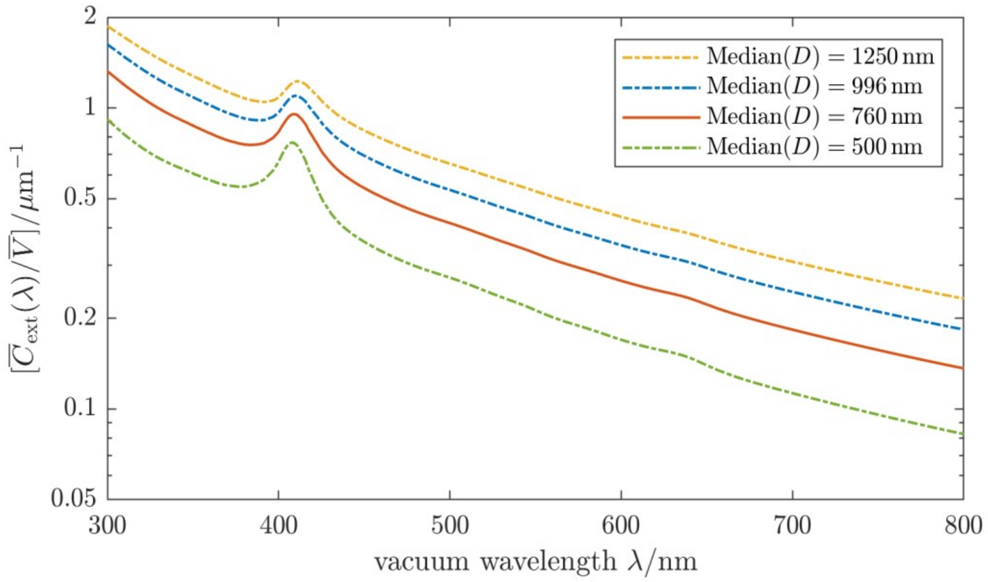

Figure S2. Influence of median particle size on the volume-specific extinction cross section for HbMPs of given relative size distribution width σ_D = 25.8% and composition. For the calculations we used the mass concentrations of 120 g L^{-1} Hb and 130 g L^{-1} Gl/HSA) and an RI corresponding to 100% metHb.

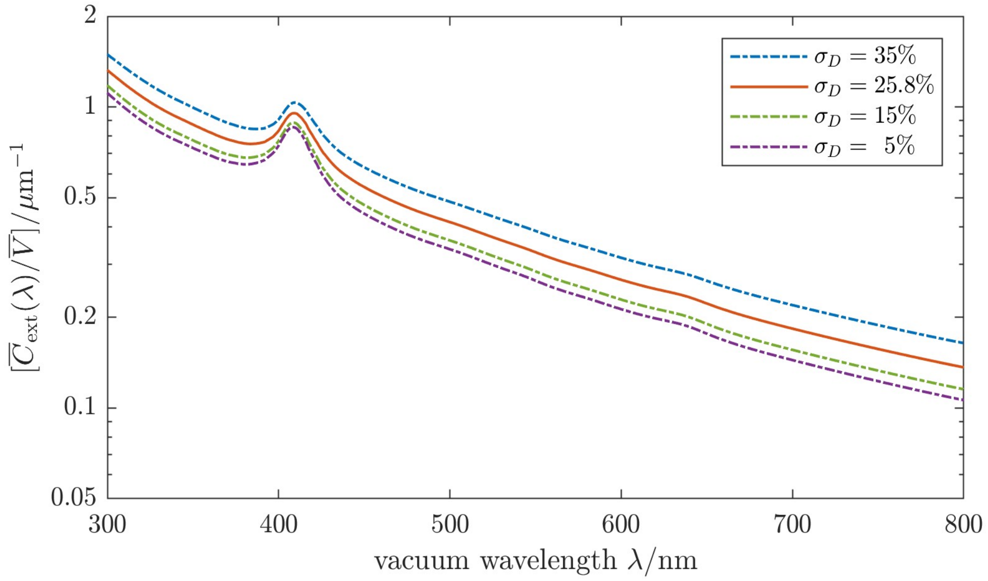

Figure S3. Influence of distribution width parameter σ_D of the log-normal particle size distribution on the volume-specific extinction cross section of HbMPs at fixed median D_{median} = 760 nm.

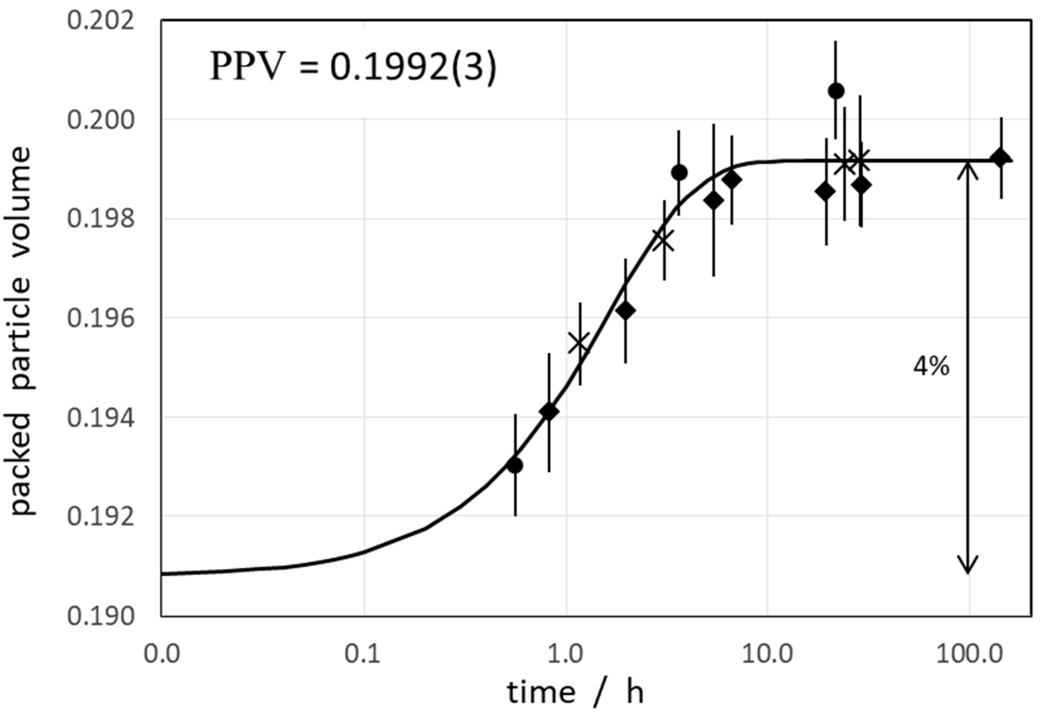

Figure S4. Packed particle volume (PPV) or solid fraction of the HbMP stock suspension as function of time. The relative centrifugation force RCF amounted to 6250 g, the centrifugation time was 21 min. The three symbols (filled circle, diamond and cross) characterize the three different persons involved in the measurements. Data were fitted by a dose response function.

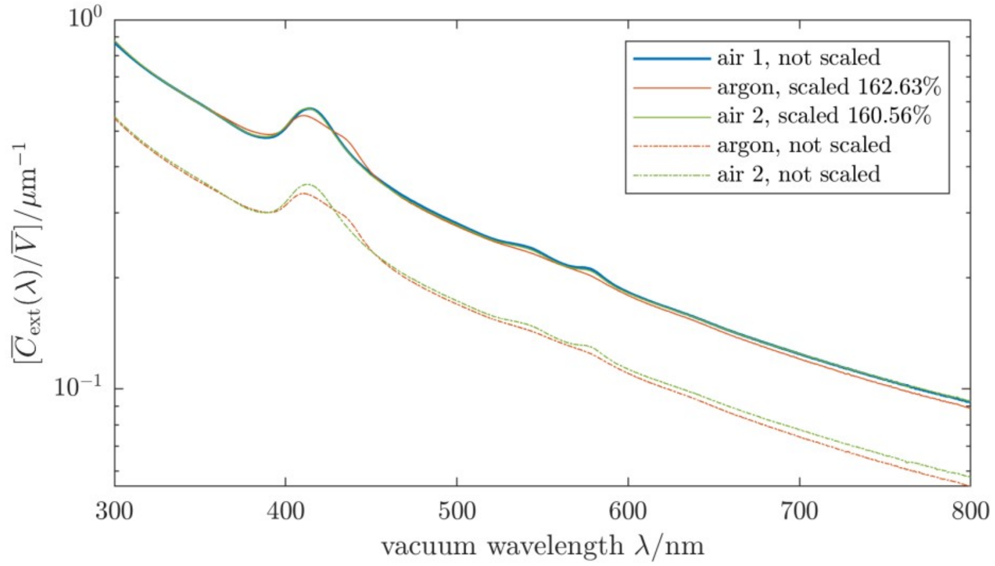

Figure S5. Oxygenation – deoxygenation and effect of particle loss. "air 1" denotes the measurement before bubbling with argon, "argon" denotes the measurement directly after and "air 2" the measurement after the sample was re-exposed to air. The latter two curves were re-scaled to have the lowest deviation from curve "air 1".

RI determination of HSA. Literature values are available for the real RI increment of HSA in aqueous solutions. However, these were either determined using white light or a single wavelength (typically the sodium D-line at 589 nm). Hence, we determined the wavelength-dependence of the real part of the RI increment by own measurements. We used a HSA infusion solution (Human Albumin 200 g L^{-1} Baxalta Infusionslösung) as the sample, which contains proteins from human plasma, of which at least 95% are albumin (HSA).

Firstly, absorption spectra were recorded with a Cary6000i UV-Vis-NIR spectrophotometer (Agilent) using multiple dilutions with ultrapure water in pair-matched 10mm cuvettes. This yields the imaginary part $\gamma_{\mathrm{HSA}}(\lambda)$ of the RI increment. At wavelengths higher than 700 nm, the absorbance is too low to be measured for the undiluted 200 g / L solution in a 10 mm cuvette. Here, we set $\gamma_{\mathrm{HSA}}(\lambda) = 0$ in the simulations (compare Figure 5). Furthermore, $\gamma_{\mathrm{HSA}}(\lambda)$ is significantly lower than $\gamma_{\mathrm{Hb}}(\lambda)$ everywhere in the spectral range of 300 nm to 800 nm. The $\gamma(\lambda)$ – curves come close to each other in the UV below 300 nm, where the spectrum of the HSA infusion solution also exhibits a significant absorbance contribution due to its content of 16 mmol/L sodium acetyltryptophanate, making up an estimated 50% of the total absorbance at 279 nm (the absorbance peak of the amino acid tryptophan). However, this is outside the spectral range under consideration. Hence, the error due to the presence of sodium acetyltryptophanate has no significant effect on the simulation results.

The real part of the spectral RI increment $\alpha_{\mathrm{HSA}}(\lambda)$ was determined using the same measurement setup as for the extinction measurements for the HbMPs. To this end, quasi-monodisperse polystyrene beads with 2.539 µm nominal diameter (Lot. PS-ST-L2552, microparticles GmbH, Germany) were suspended in the 200 g L^{-1} HSA solution at different

particle concentrations and the collimated transmittance was measured. Given the spectral RI of the particles, obtained from an extinction measurement in pure water[S1], the real part of the spectral RI of the suspending fluid (i.e., the HSA solution) can be obtained from these measurements. For this purpose, the method described in Ref. S1 was modified[S2]. The fact that the HSA solution absorbs light in UV limits the wavelength range available for transmittance measurements to $\lambda \geq 300$ nm, but otherwise does not hamper the data analysis which is based on the assumption of a non-absorbing fluid[S2]. Hence, the RI of the aqueous HSA solution is

$$n(\lambda) = n_{\mathrm{H_2O}}(\lambda) + \beta_{\mathrm{HSA}}\ \alpha_{\mathrm{HSA}}(\lambda) \quad . \tag{S1}$$

For the model fit of the measured $\bar{C}_{\mathrm{ext}}(\lambda)$ the spectral dependence of the RI increment $\alpha_{\mathrm{HSA}}(\lambda)$ was expressed by a 2-term Cauchy equation, i.e.

$$\alpha_{\mathrm{HSA}}(\lambda) = B + \frac{C}{\lambda^2}. \quad . \tag{S2}$$

The result for the coefficients is $B = 0.180\ \mathrm{mL\ g^{-1}}$ and $C = 3.2 \times 10^{-3} \mathrm{mL\ g^{-1} \mu m^{-2}}$ for $300\ \mathrm{nm} \leq \lambda \leq 1100\ \mathrm{nm}$. The estimated uncertainty of $\alpha_{\mathrm{HSA}}(\lambda)$ is $u(\alpha_{\mathrm{HSA}}(\lambda)) = 0.008\ \mathrm{mL\ g^{-1}}$. For verification purposes, the RI was also determined with an Abbe refractometer at 590 nm, which resulted in $0.190(4)\ \mathrm{mL\ g^{-1}}$ compared to $0.188(8)\ \mathrm{mL\ g^{-1}}$ from the Cauchy equation. Both values agree with each other as well as with literature values[S3].

As already stated in Section Model for Optical Properties of HbMPs of the paper, we take the optical properties of HSA for both, HSA and (heme-free) globin, since the increments of the real and imaginary parts $\alpha(\lambda)$ and $\gamma(\lambda)$ for globin are unknown but expected to be very similar to HSA. Compared to Hb, globins do not have a heme group causing a strong absorption associated

with strong variations of the complex RI. In addition, the molecular masses of the two globular proteins are similar.

(S1) Gienger, J.; Bär, M. ; Neukammer, J. Extinction spectra of suspensions of microspheres: determination of the spectral refractive index and particle size distribution with nanometer accuracy. *Applied Optics* **2018**, Vol. 57, 344-355.

(S2) Gienger, Jonas. Determination of optical and geometrical properties of blood cells and microparticles from light scattering measurements. *PhD thesis* **2019**, Technische Universität Berlin.

(S3) Barer, R. and Joseph**, S.** Refractometry of Living Cells: Part I. Basic Principles. *Quarterly Journal of Microscopical Science* **1954**, Vols. s3-95, 32, pp. 399-423.

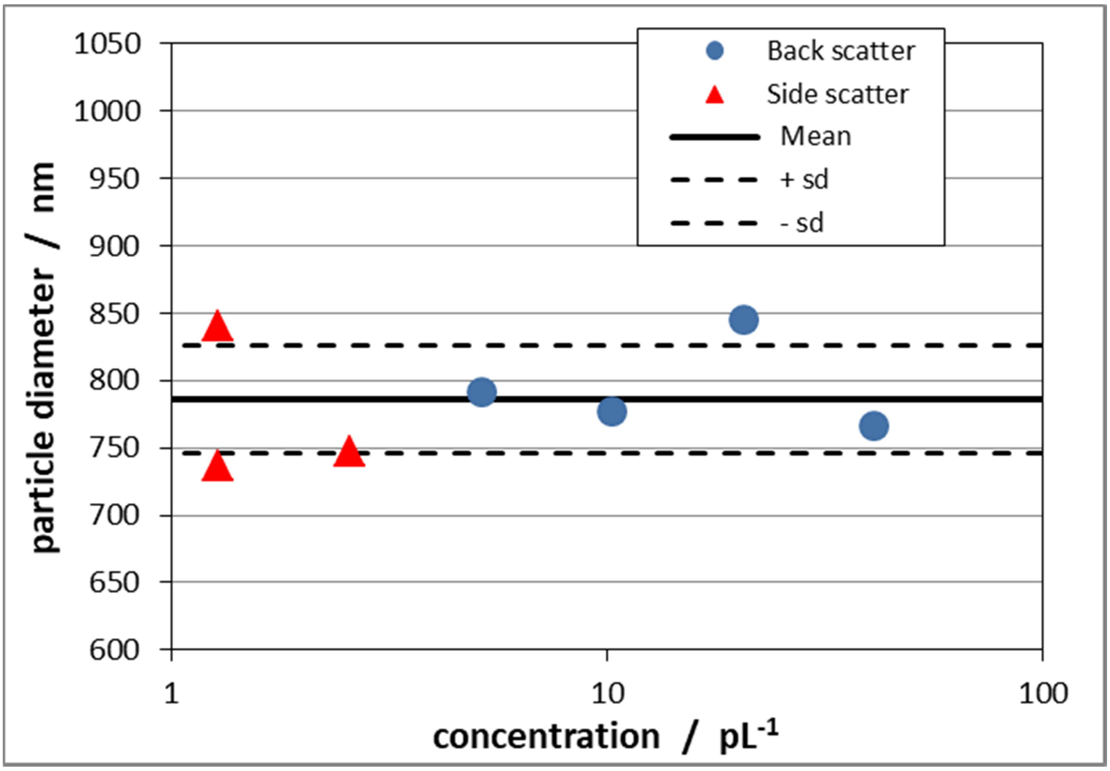

Figure S6. Particle diameter of HbMPs measured by DLS for different concentrations using back scatter (blue dots) and side scatter (red triangles). The mean value is plotted as black straight line and the standard deviation is included as dashed lines.

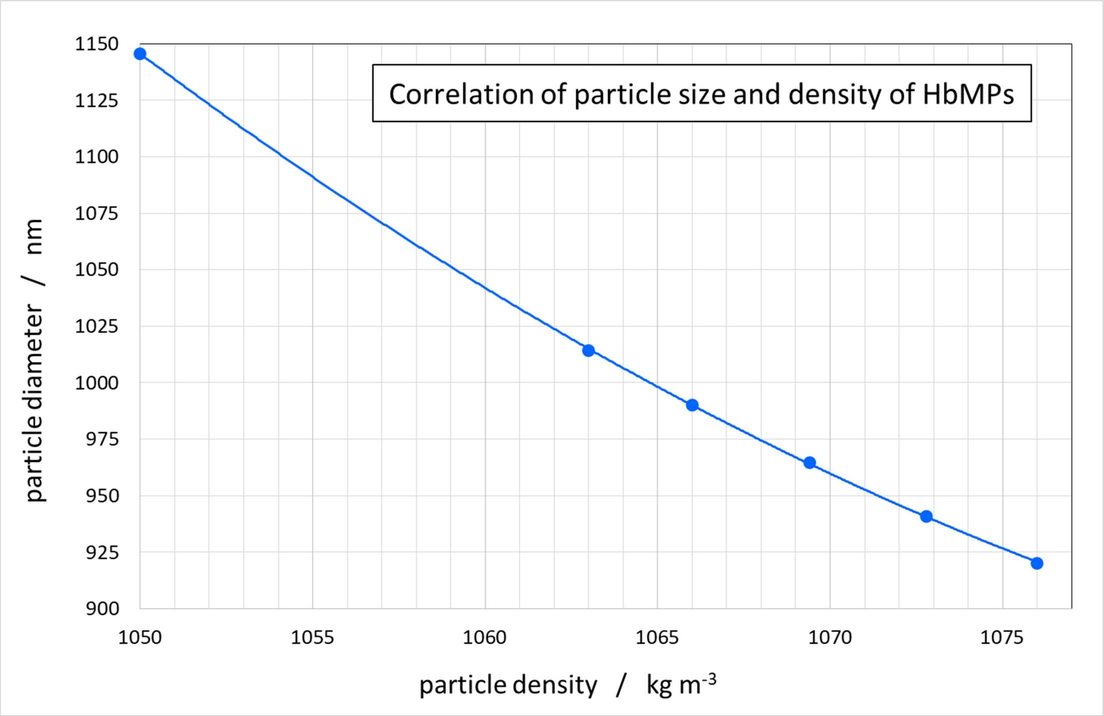

Figure S7. Correlation of the results for the particle size calculated from AC sedimentation measurements and the density of the HbMPs in the region of the values derived by the mechanical oscillator method. The line is interpolated by a polynomial equation.

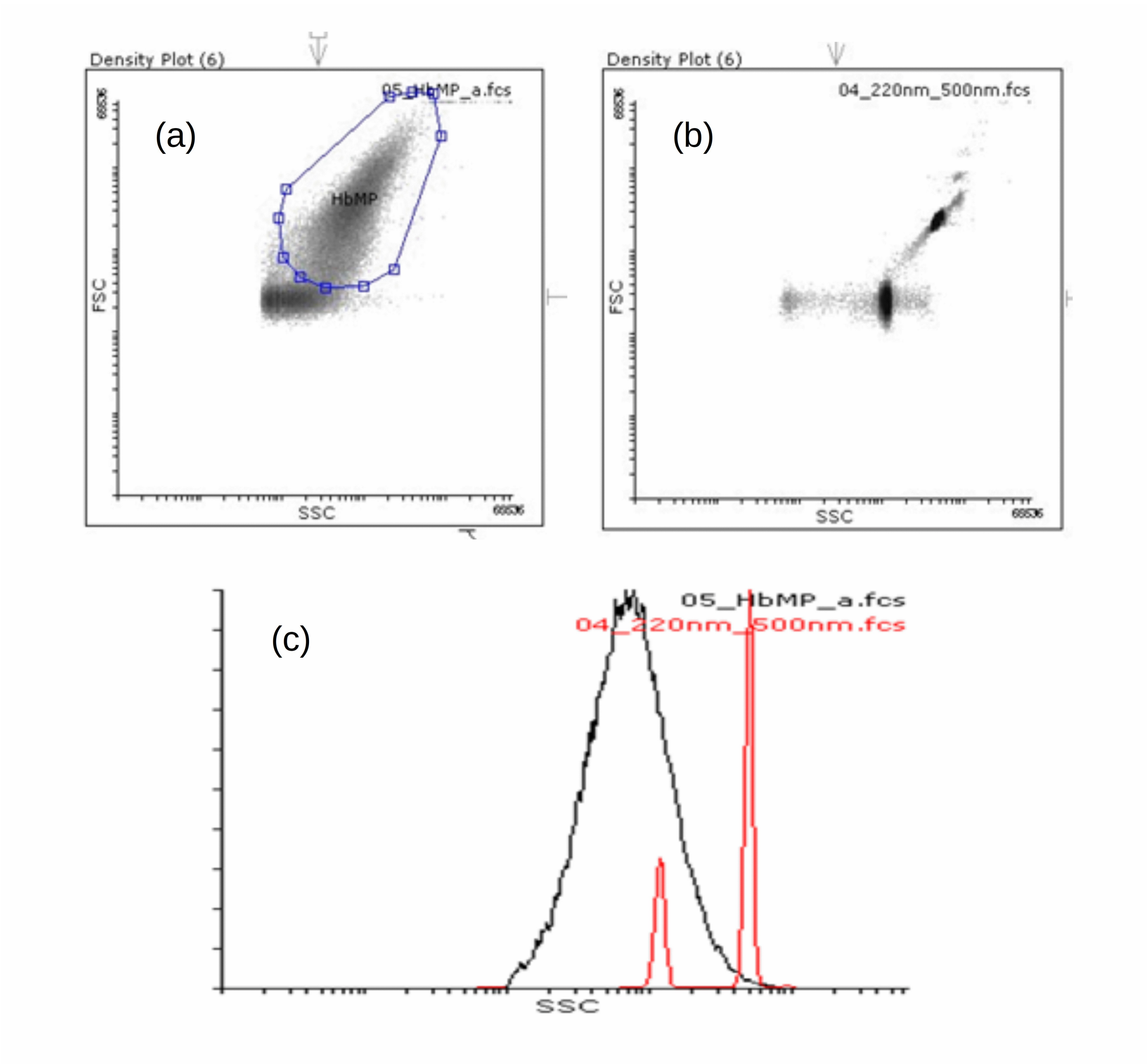

Figure S8. Flow cytometric measurements of HbMPs and polystyrene microspheres. Dot plots of signal intensities for forward light scatter versus side scatter for HbMPs (a) and polystyrene microparticles (b). The cluster of HbMPs is gated to show only the population of HbMPs in the (c) SSC histogram together with signals originating from monodisperse polystyrene particles with diameters of 220 nm and 500 nm, respectively.

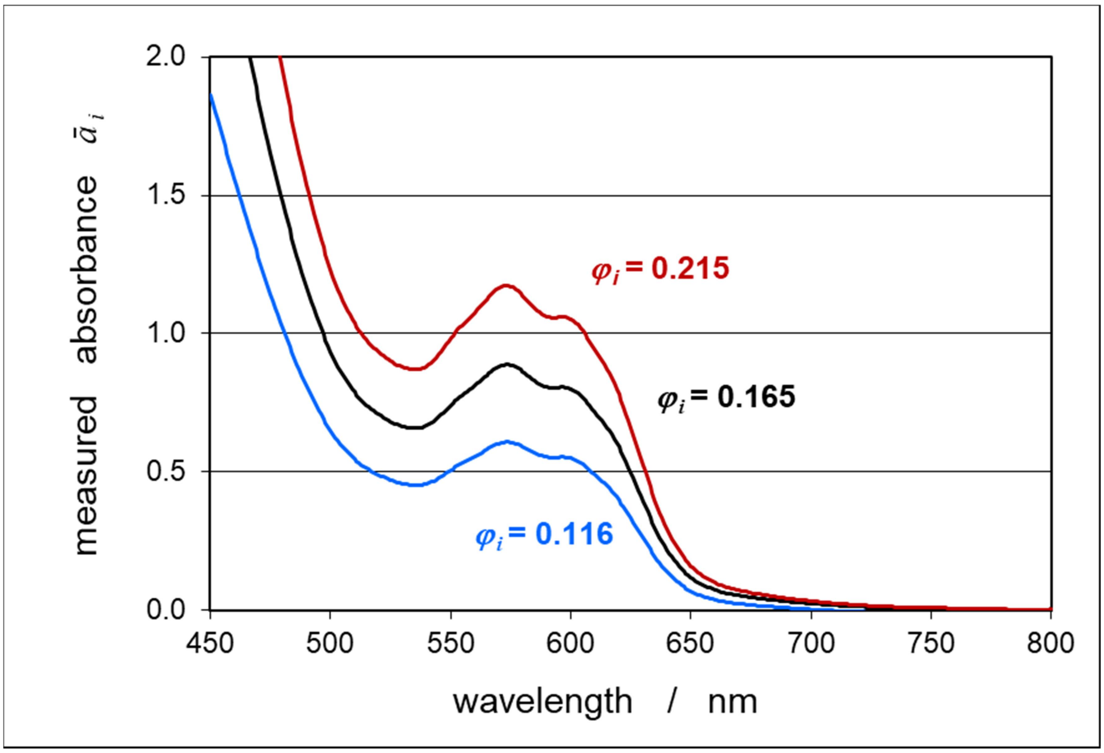

Figure S9. Absorption spectra of the HbMP suspension resulting from different dilutions. The respective volume fractions φ_i of the HbMP suspension in the measurement sample are included in the Figure. HbMPs were digested by a Pronase solution and converted by AHD reagents in a stable end product, i.e. hematin.
